# How Pragmatic Are Sarcopenia Intervention Studies? A Systematic Review

**DOI:** 10.1002/jcsm.70181

**Published:** 2026-01-22

**Authors:** Sophie Van Heden, Zoubayda Baoubbou, Dolores Sanchez‐Rodriguez, Yoke Mun Chan, Charlotte Beaudart

**Affiliations:** ^1^ Public Health Aging Research & Epidemiology (PHARE) Group, Research Unit in Clinical Pharmacology and Toxicology (URPC), Department of Biomedical Sciences, Namur Research Institute for Life Sciences (NARILIS), Faculty of Medicine, University of Namur Namur Belgium; ^2^ Geriatrics Department Brugmann University Hospital, Université Libre de Bruxelles Brussels Belgium; ^3^ Rehabilitation Research Group, Hospital del mar Research Institute Barcelone Spain; ^4^ Department of Dietetics, Faculty of Medicine and Health Sciences University Putra Malaysia Serdang Malaysia; ^5^ Malaysian Research Institute on Ageing University Putra Malaysia Serdang Malaysia

**Keywords:** interventional, pragmatism, PRECIS‐2, RCTs, sarcopenia

## Abstract

**Background:**

Sarcopenia is an age‐related muscle disease often accompanied by comorbidities, mobility issues and cognitive decline, which can limit treatment adherence in older adults. Owing to the reversible nature of sarcopenia, there has been a growing number of randomized controlled trials conducted in recent years. Yet, many randomized controlled trials (RCTs) are conducted under ideal conditions (explanatory trials), limiting their real‐world applicability. In contrast, pragmatic trials aim to better reflect the complexities of clinical practice.

**Objective:**

This study is aimed at assessing the level of pragmatism in current sarcopenia RCTs and identifying design gaps to further improve the clinical relevance and feasibility of future trials in the real world.

**Methods:**

A systematic review was conducted on MEDLINE (via Ovid), Embase and Cochrane Central Register of Controlled Trials (PRISMA guidelines; PROSPERO: CRD42024571027). Eligible studies included RCTs on sarcopenia treatment using a consensus definition and published until March 2024. The PRECIS‐2 tool was used to assess the level of pragmatism of these RCTs across nine standard domains (eligibility, recruitment, setting, organization, flexibility of delivery, flexibility of adherence, follow‐up, primary outcome and primary analysis), with an additional ‘control’ domain. The total PRECIS‐2 score was calculated, and subgroup analyses were conducted by intervention type, geographical location, sample size, study duration and sarcopenia definition. A higher PRECIS‐2 score indicates greater trial pragmatism. The risk of bias was assessed using the Cochrane Risk of Bias 2.0 tool.

**Results:**

Of the 3985 references reviewed, 54 RCTs met the inclusion criteria. The mean PRECIS‐2 score across its 10 domains was 2.93 (SD 1.30), reflecting a balance of explanatory and pragmatic characteristics. Organization, recruitment and primary outcome were identified as the most pragmatic domains, whereas eligibility, adherence and follow‐up were the most explanatory. Subgroup analyses revealed that geographical location and sarcopenia definitions impacted significantly the overall PRECIS‐2 score. More precisely, studies conducted in Asia achieved higher pragmatism scores, with significant differences in setting (*p* = 0.029), follow‐up (*p* = 0.014) and control (*p* = 0.042) domains. Studies using Asian sarcopenia criteria (e.g., AWGS) were also more pragmatic, particularly in the eligibility (*p* = 0.031) and control (*p* = 0.005) domains.

**Conclusion:**

This systematic review reveals a persistent gap between explanatory and pragmatic designs in sarcopenia trials. Despite growing research, few studies reflect real‐world conditions. Key domains like eligibility, adherence and follow‐up remain overly controlled. Greater pragmatism is needed to ensure future trials yield evidence that is both robust and clinically applicable.

## 1 Introduction

Sarcopenia is a progressive disease characterized by the loss of muscle strength and mass, with physical performance used to assess its severity, as defined by the European Working Group on Sarcopenia in Older People (EWGSOP2) [1, 2]. Since 2016, it has been officially recognized as a distinct clinical entity, with the allocation of a specific ICD‐10‐CM code [3, 4].

The Global Leadership Initiative on Sarcopenia (GLIS) was recently established to harmonize existing definitions and develop a universally accepted standard [5]. This effort is particularly critical given that sarcopenia affects more than 10% of adults aged 65 years and older [6, 7], and is associated with numerous health consequences, including decreased physical performance and mobility, impaired ability to perform daily living activities, reduced quality of life, increased risk of falls and fractures, hospitalizations, greater likelihood of admission to long‐term care facilities and higher mortality rates [8, 9].

Sarcopenia is considered a potentially reversible disease [10]. This underscores the importance of interventional research aimed at identifying effective therapeutic strategies. To date, recommended treatments primarily focus on exercise, both endurance and resistance training [11, 12] and nutritional interventions, such as supplementation with vitamin D, protein, polyunsaturated fatty acids, antioxidants and adequate caloric intake [12]. The combination of physical activity and nutritional strategies currently represents the most effective approach [10, 13, 14]. No pharmacological treatment is yet approved for this indication, although several promising molecules, including apelin and irisin, are under investigation [15, 16].

Clinical trials investigating sarcopenia face numerous methodological and practical challenges. Older adults, and particularly older adults with sarcopenia, frequently present with multiple comorbidities [17, 18], geriatric syndromes and functional [8, 9], or cognitive impairments [19, 20, 21, 22] that may limit participation and adherence to highly controlled trial protocols. As sarcopenia is frequently accompanied by multimorbidities, strictly explanatory randomized controlled trials (RCTs) may fail to capture the complex clinical realities of these patients. Consequently, such trials often exclude the populations most affected, thereby limiting the external validity and real‐world applicability of their findings [23]. These challenges underscore the importance of pragmatic trial designs, which better reflect routine clinical practice and ensure that study results are directly relevant to those most affected by sarcopenia [23].

While pragmatic clinical trials are specifically designed to evaluate the effectiveness of interventions under real‐world conditions by accommodating clinical diversity, flexible protocols and outcomes relevant to both patients and healthcare providers [23, 24], much of the current evidence on sarcopenia still stems from highly controlled trial protocols. This limits the generalizability and practical value of research findings for routine clinical practice. To date, there is an information gap regarding the level of pragmatism along the pragmatic–explanatory continuum in clinical trials on sarcopenia. Bridging this gap is essential for enhancing the relevance and applicability of research findings to everyday clinical practice and for developing tailored therapeutic interventions that address the unmet needs of older adults with sarcopenia who face complex, real‐world health challenges.

The aim of this study is to assess the level of pragmatism within the pragmatic–explanatory continuum in published RCTs investigating interventions for managing sarcopenia in older adults. This approach seeks to identify methodological gaps and provide recommendations to enhance the clinical relevance and feasibility of future trials in real‐world settings.

## 2 Methods

### 2.1 Generality

This systematic literature review was carried out in accordance with the Preferred Reporting Items for Systematic Reviews and Meta‐Analyses (PRISMA) [25], ensuring methodological rigour and transparency. The completed PRISMA checklist is available in appendix (Table S1). The study protocol has been registered in the international PROSPERO database (registration number: CRD42024571027).

To ensure transparency and facilitate further exploration, all relevant documents and supplementary materials have been made publicly available on the Open Science Framework (OSF) platform (https://osf.io/bx429/).

### 2.2 Search Strategy and Study Selection

A comprehensive literature search was performed in March 2024 across Medline (via Ovid), Embase and the Cochrane Central Register of Controlled Trials (CENTRAL), using a predefined search strategy detailed in appendix (Table S2), to identify all RCTs specific to sarcopenia. Additional sources included manual screening of reference lists from relevant publications, citation tracking via Web of Science and consultations with domain experts. Previous systematic reviews and meta‐analyses on sarcopenia intervention studies were also reviewed to identify further eligible studies.

All studies retrieved from electronic databases and manual searches were imported into Covidence, a web‐based systematic review management platform designed to facilitate efficient screening, data extraction and other literature reviews. The study criteria for this systematic review are presented in Table 1.

**TABLE 1:** Inclusion criteria of the systematic review (population/concept/context, PCC).

Population	Older adults aged 60 years of age or over. Studies were included if the mean sample age was above 60 years, or results were reported separately for people aged 60 years or over. Sarcopenia consensus definition includes at least a measurement of two key parameters of sarcopenia (e.g., muscle mass + (muscle strength or physical function). The following definitions were accepted: 1) European Working Group on Sarcopenia in Older People version 1, 2) European Working Group on Sarcopenia in Older People version 2, 3) Foundation for the National Institutes of Health Sarcopenia Project, 4) Asian Working Group on Sarcopenia, 5) Society on Sarcopenia, Cachexia and Wasting Disorders, 6) International Working Group on Sarcopenia, and 7) Sarcopenia Definitions and Outcomes Consortium.
Concept	Randomized controlled trial, aiming at the management of sarcopenia using any type of intervention (exercises, nutrition, combined exercises & nutrition, pharmacological treatments, and other interventions)
Context	*Inclusion criteria:* Articles published until March 2024 (date when the last bibliographic search is consulted). Only studies written in English were considered eligible for inclusion [26]. Only full‐text, peer‐reviewed publications in indexed journals were included. *Exclusion criteria:* Case reports, congress abstracts, reviews, protocols, and letters to the editors (unless they contain original data).

**FIGURE 1:**
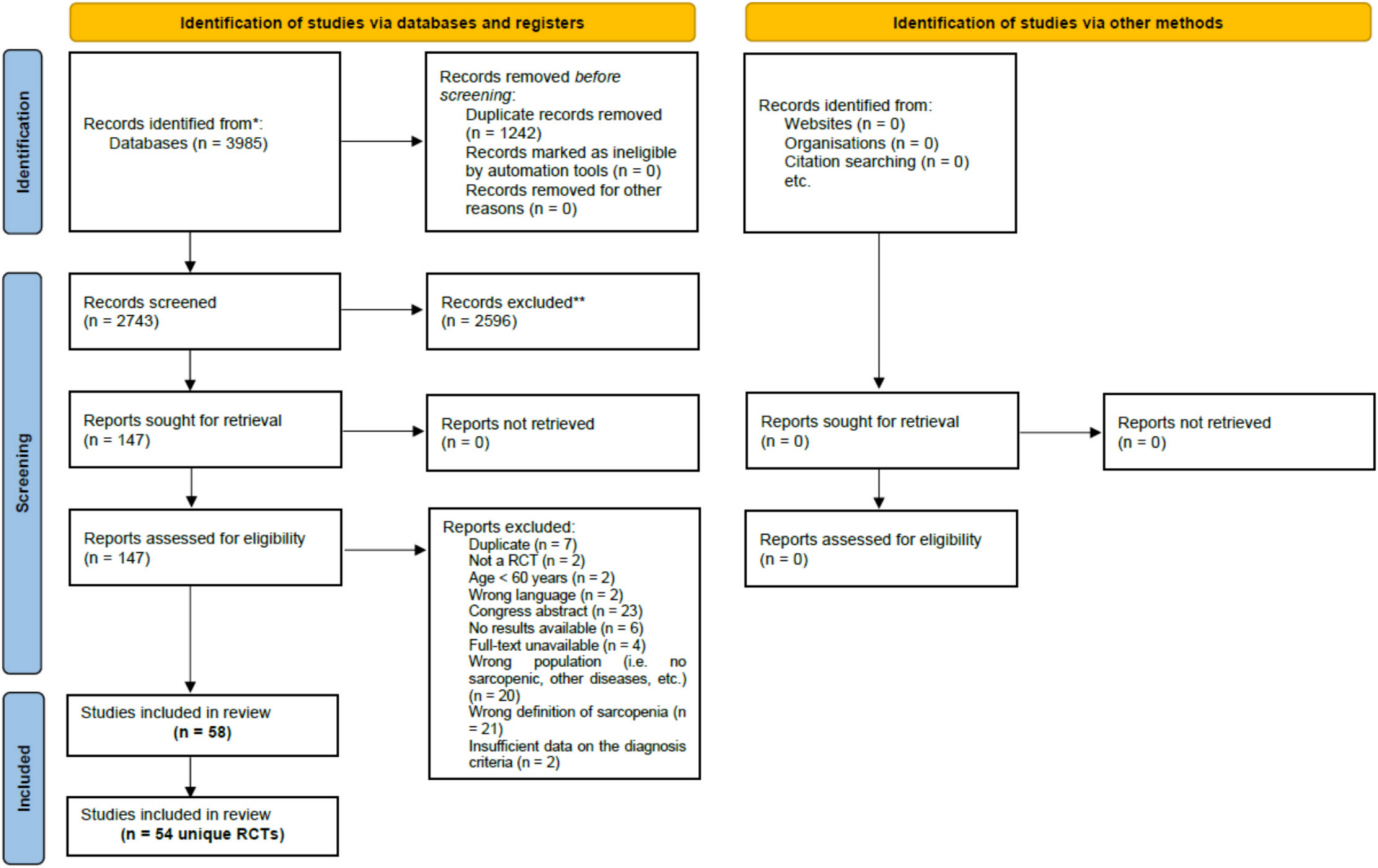
PRISMA 2020 flow diagram.

All retrieved records were independently screened by reviewers (CB, DSR and YMC) based on titles and abstracts, followed by full‐text assessments to determine eligibility. Each study required evaluation by at least two reviewers, and any disagreements were resolved through discussion to reach consensus. The study selection process is outlined in the PRISMA flow diagram (Figure 1), with reasons for exclusion clearly documented and reported.

### 2.3 Data Extraction

The extracted data were recorded in a standardized Excel file that had been pretested on a sample of five studies. Data extraction was performed independently by two reviewers (SVH and ZB) to ensure accuracy and minimize bias. The extracted data included general study information, such as study design, study sample, study duration, geographical location, inclusion criteria, exclusion criteria, recruitment details, organization, type of intervention and arms, details of intervention group and control group, adherence, follow‐up, type of analysis (per protocol or intention‐to‐treat) and primary outcome.

If a RCT was included in several studies, only the first published study was considered in the statistical analysis (since the PRECIS‐2 pragmatic domains are identical for a single RCT) to avoid over‐representation of a single trial.

### 2.4 Pragmatism Assessment

Pragmatism was assessed using the PRagmatic Explanatory Continuum Indicator Summary (PRECIS)‐2 tool [27, 28]. It was developed to help trialists consider where their studies lie on the pragmatic/explanatory continuum during the design phase. The tool can also be used in a retrospective approach to address the level of pragmatism of published clinical trials [29]. PRECIS‐2 includes nine domains: eligibility, recruitment, setting, organization, flexibility in delivery, flexibility in adherence, follow‐up, primary outcome and primary analysis. In addition to the original PRECIS‐2 domains, this study incorporated an extra domain assessing the design of control groups. Although this is not formally part of the PRECIS‐2 tool, Zwarenstein et al. [29], who were involved in the refinement of the original PRECIS into PRECIS‐2 [30], have suggested the inclusion of a control‐specific domain to better assess whether control conditions align with pragmatic principles, particularly in retrospective evaluations of clinical trials. This adaptation was made to enhance the tool's ability to assess pragmatism in the context of sarcopenia trials.

Pragmatism assessment was performed independently by two reviewers (SVH and ZB). Before starting the full evaluation, both reviewers jointly assessed two sample trials to calibrate their understanding of the tool and refine the application of the scoring criteria. Any disagreements were resolved through consensus, with the option of consulting a third party (CB) if necessary to reach a final decision. Each domain scores from 1 to 5, and a higher PRECIS‐2 score indicates greater pragmatism. A scoring table (Table 2) was developed based on the PRECIS‐2 guidelines [31], existing literature [32, 33] and the critical evaluation of the research team. This scoring methodology was applied to each study.

**TABLE 2:** PRECIS‐2 scoring of included randomized controlled trials, based on guidelines, literature, and critical evaluation of researchers.

PRECIS‐2 domains	Score 1	Score 2	Score 3	Score 4	Score 5
**Eligibility** To what extent are the participants in the trial similar to those who would receive this intervention if it was part of usual care? For example, score 5 for very pragmatic criteria essentially identical to those in usual care; score 1 for a very explanatory approach with lots of exclusions (e.g., those who do not comply, respond to treatment, or are not at high risk for primary outcome, are children or elderly), or uses many selection tests not used in usual care.	More than five inclusion criteria, with very specific requirements for participants (e.g., exclusion based on comorbidities, medication use, or high‐risk conditions).	Several exclusion criteria that limit participation to specific populations or conditions (e.g., need for laboratory results or pre‐enrolment tests).	Few inclusion criteria, with 1–2 specific exclusions for common conditions or treatments.	Minimal exclusion criteria focus only on conditions directly interfering with the intervention or outcomes.	Broad criteria reflecting real‐world applicability, excluding only for safety or ethical reasons.
**Recruitment** How much extra effort is made to recruit participants over and above what that would be used in the usual care setting to engage with patients? For example, score 5 for very pragmatic recruitment through usual appointments or clinic; score 1 for a very explanatory approach with targeted invitation letters, advertising in newspapers, radio plus incentives and other routes that would not be used in usual care.	Recruitment involves multiple methods such as mass advertising, invitations, incentives, or outreach campaigns not used in routine care.	Recruitment involves active outreach through advertisements, emails, or targeted efforts in specific regions.	Combination of standard clinical referrals and limited advertising efforts.	Recruitment focuses on patients attending regular clinics or hospitals with minimal advertising.	Recruitment occurs exclusively through routine clinical care, as patients naturally seek treatment.
**Setting** How different is the setting of the trial and the usual care setting? For example, score 5 for a very pragmatic choice using identical settings to usual care; score 1, for a very explanatory approach with only a single centre, or only specialized trial or academic centres.	Single, highly specialized centre or research‐focused institution, not representative of usual care settings.	Trials conducted in multiple specialized centres, often with a research‐oriented approach.	Conducted in a mix of primary and secondary care facilities, with moderate representation of usual care.	Conducted across several community hospitals, primary care clinics, or similar routine settings.	Fully representative settings, identical to those used in routine care (e.g., multiple general practices or hospitals).
**Organization** How different are the resources, provider expertise and the organization of care delivery in the intervention arm of the trial and those available in usual care? For example, score 5 for a very pragmatic choice that uses identical organization to usual care; score 1 for a very explanatory approach if the trial increases staff levels, gives additional training, require more than usual experience or certification and increase resources.	Requires highly trained personnel, advanced equipment, and significant adjustments to usual workflows.	Demands moderate additional resources such as specialized training or advanced diagnostic tools.	Standard resources with some minor additions, such as short training sessions for staff.	Minimal organizational changes, with the intervention integrated into existing workflows without disrupting usual care.	No additional resources, training, or adjustments required, intervention aligns perfectly with routine care.
**Flexibility – Delivery** How different is the flexibility in how the intervention is delivered and the flexibility likely in usual care? For example, score 5 for a very pragmatic choice with identical flexibility to usual care; score 1 for a very explanatory approach if there is a strict protocol, monitoring and measures to improve compliance, with specific advice on allowed co‐interventions and complications.	Intervention delivered under strict protocols with no room for adaptation or individualization (e.g., strict dosage, timing, or method enforcement).	Delivery is standardized but allows for small adjustments to accommodate patient needs.	Moderately flexible delivery, allowing practitioners to adapt to individual patient needs within predefined limits.	Broadly individualized intervention with few constraints, enabling customization based on usual care practices.	Fully flexible delivery, mirroring real‐world clinical scenarios with no active monitoring or restrictions.
**Flexibility – Adherence** How different is the flexibility in how participants must adhere to the intervention and the flexibility likely in usual care? For example, score 5 for a very pragmatic choice involving no more than usual encouragement to adhere to the intervention; score 1 for a very explanatory approach that involves exclusion based on adherence, and measures to improve adherence if found wanting. In some trials, e.g., surgical trials where patients are being operated on or Intensive Care Unit trials where patients are being given IV drug therapy, this domain is not applicable as there is no compliance issue after consent has been given, so this score should be left blank.	Adherence is monitored rigorously through frequent follow‐ups, direct supervision, or detailed tracking methods (e.g., biological measures, daily logs).	Adherence is supported through regular monitoring using structured tools (e.g., attendance logs, patient diaries).	Moderate adherence monitoring, with occasional reminders or patient feedback.	Minimal adherence monitoring, with rare follow‐ups or passive checks such as medical record reviews.	Adherence is not actively monitored, reflecting usual care where patients self‐manage participation and compliance.
**Follow‐up** How different is the intensity of measurement and follow‐up of participants in the trial and the likely follow‐up in usual care? For example, score 5 for a very pragmatic approach with no more than usual follow up; score 1 for a very explanatory approach with more frequent, longer visits, unscheduled visits triggered by primary outcome event or intervening event, and more extensive data collection.	Follow‐up is highly intensive, with numerous visits, detailed evaluations, and frequent data collection over short intervals.	Regular follow‐ups involve a variety of tests or measurements, moderately exceeding usual care intensity.	Follow‐up intervals are aligned with standard care but include additional data points for analysis.	Minimal follow‐up aligned with critical intervention points, without unnecessary visits or measurements.	Follow‐up is limited to baseline and final assessments or relies on routine care records without additional visits.
**Primary outcome** To what extent is the trial's primary outcome relevant to participants? For example, score 5 for a very pragmatic choice where the outcome is of obvious importance to participants; score 1 for a very explanatory approach using a surrogate, physiological outcome, central adjudication or use assessment expertise that is not available in usual care, or the outcome is measured at an earlier time than in usual care.	Outcomes focus on surrogate or laboratory markers requiring specialized equipment or expertise, with minimal relevance to patient priorities.	Outcomes are technical but have some indirect relevance to health or quality of life (e.g., composite measures).	Outcomes moderately relevant to patients, reflecting clinically significant improvements but not directly prioritized by them.	Outcomes directly impactful, highlighting measurable health benefits or improvements in quality of life.	Outcomes are highly relevant and prioritized by patients, directly reflecting their experiences and expectations (e.g., functional improvement, symptom relief).
**Primary analysis** To what extent are all data included in the analysis of the primary outcome? For example, score 5 for a very pragmatic approach using intention to treat with all available data; score 1 for a very explanatory analysis that excludes ineligible post‐randomization participants, includes only completers or those following the treatment protocol	Analysis excludes post‐randomization participants with protocol violations or missing data, focusing solely on a per‐protocol approach.	/	/	Nearly all participants randomized are included in the analysis, with minimal exclusions based on predefined rules.	Intention‐to‐treat analysis includes all participants as randomized, regardless of adherence or data completeness.
**Control** How similar is the comparator in the trial to what participants would receive in usual care? For example, score 5 for a very pragmatic approach using an active treatment or usual care as the comparator; score 1 for a very explanatory approach using a placebo or a blinded comparator that would not normally be offered in routine practice.	Control group receives a placebo (e.g., sham treatment) designed to mimic the intervention without therapeutic effects.	Control groups receive minimal intervention, such as educational materials or monitoring.	Control group receives basic care reflective of usual clinical practice but lacks intervention equivalence.	Control group receives enhanced care or structured interventions, but without matching the experimental treatment intensity.	Control group receives standard care identical to routine clinical practice, ensuring real‐world relevance.

### 2.5 Risk of Bias Assessment

The quality of individual RCTs was evaluated using the Cochrane Risk of Bias 2.0 tool, applied independently by two reviewers (YMC and SVH). Any disagreements were resolved through consensus, with the option of consulting a third party (DSR) if necessary to reach a final decision [34].

### 2.6 Statistical Analysis

Descriptive statistics were used to summarize the PRECIS‐2 scores across domains. Specifically, the mean and standard deviation (SD) were calculated for each domain to provide an overall view of trial pragmatism. To enhance interpretability, graphical representations of PRECIS‐2 profiles were created using radar plots (‘PRECIS‐2 wheels’), allowing for a visual assessment of the degree of pragmatism across RCTs. Subgroup analyses were conducted to explore variations in PRECIS‐2 scores according to key study characteristics. Comparisons were made based on intervention type (exercise, nutrition, combined intervention, pharmacological treatments and other unclassified interventions), geographical location (continents), sample size (< 75 people, > 75 people—median of included RCTs), study duration (≤ 12 weeks, > 12 weeks—median of included RCTs), and definitions of sarcopenia (EWGSOP, AWGS, and other). Inter‐rater reliability of PRECIS‐2 scoring was assessed using intraclass correlation coefficients (ICC) [35].

Statistical comparisons between groups were performed using Student's *t*‐test for pairwise comparisons and one‐way ANOVA for comparisons involving more than two groups. Two sensitivity analyses were carried out to assess the quality of our results: one excluding the ‘control’ domain, and the other excluding studies with a high risk of bias. All statistical analyses were performed using R software. The data set and study codebook are available on Open Science Framework (https://osf.io/bx429/).

## 3 Results

After removing duplicates, a total of 2743 records were screened based on title and abstract. Of these, 147 articles underwent full‐text evaluation to assess eligibility. Ultimately, 54 RCTs met the inclusion criteria and were retained for the systematic review (Figure 1, PRISMA flowchart). Detailed reasons for the exclusion of the remaining studies are provided in the supplementary material (Table S3).

### 3.1 Studies Description

Table 3 presents the characteristics of the 54 included RCTs. These RCTs, published between 2012 and 2024, utilized various definitions of sarcopenia, with EWGSOP 2010 being the most frequently applied (20 RCTs, 37.0%), followed by AWGS 2014 (19 RCTs, 35.2%). Of the RCTs included in the review, 16 (29.6%) had fewer than 50 participants, 21 (38.9%) had between 51 and 100 participants and 17 (31.5%) had more than 100 participants. Most studies lasted between 10 and 20 weeks (24 RCTs, 44.4%), followed by studies lasting between 20 and 30 weeks (14 RCTs, 25.9%). In terms of interventions, exercise‐based interventions were the most common (19 RCTs, 35.2%), followed by a combination of both exercise and nutrition approaches (17 RCTs, 31.5%), nutritional interventions (11 RCTs, 20.4%), pharmacological interventions (four RCTs, 7.4%) and other types of interventions (three RCTs, 5.6%). Geographically, the majority of RCTs were conducted in Asia (31 RCTs, 57.4%), followed by Europe (13 RCTs, 24.1%) and America (seven RCTs, 13.0%). The remaining three RCTs (5.6%) were from other regions, including Oceania and various countries. Most of the recruitment was community‐based (36 RCTs, 66.7%), and the majority of the studies were multicentre (33 RCTs, 61.1%). Regarding funding, 22 RCTs (40.7%) received academic funding, and 19 RCTs (35.2%) received industrial funding. A detailed description of each included RCT is available in the supplementary material (Table S4).

**TABLE 3:** Main characteristics of included RCTs evaluating treatments for sarcopenia in older adults (*n* = 54).

Characteristics	Categories	Number of RCTs (%)
Publication year	< 2015	3 (5.6%)
	2015–2019	15 (27.8%)
	2020–2024	36 (66.7%)
Definition of sarcopenia	EWGSOP	20 (37.0%)
	AWGS2014	19 (35.2%)
	FNIH	2 (3.7%)
	EWGSOP2	4 (7.4%)
	AWGS2019	5 (9.3%)
	Other definition	4 (7.4%)
Number of participants	0–50	16 (29.6%)
	51–100	21 (38.9%)
	> 101	17 (31.5%)
Duration of the study	< 10 weeks	10 (18.5%)
	10 weeks–< 20 weeks	24 (44.4%)
	20 weeks–< 30 weeks	14 (25.9%)
	> 30 weeks	6 (11.1%)
Type of intervention	Exercise	19 (35.2%)
	Nutrition	11 (20.4%)
	Exercise + nutrition	17 (31.5%)
	Pharmacological treatments	4 (7.4%)
	Other	3 (5.6%)
Geographical distribution	Asia	31 (57.4%)
	Europe	13 (24.1%)
	America	7 (13.0%)
	Other	3 (5.6%)
Research site	Hospital	7 (13.0%)
	Community	36 (66.7%)
	NR	11 (20.4%)
Number of centres	Single centre	14 (25.9%)
	Multiple centre	33 (61.1%)
	NR	7 (13.0%)
Funding	Industry	19 (35.2%)
	Academic	22 (40.7%)
	No funding/NR	13 (24.1%)
Risk of bias (Cochrane Risk of Bias 2.0)	Low risk of bias	36 (66.7%)
	Some concerns	11 (20.4%)
	High risk of bias	7 (13.0%)

Abbreviations: AWGS, Asian Working Group for Sarcopenia; EWGSOP, European Working Group on Sarcopenia in Older People; FNIH, Foundation for the National Institutes of Health Sarcopenia Project; NR, not reported; RCT, randomized controlled trial.

The risk of bias was evaluated using Cochrane Risk of Bias 2.0. For analyses according to the intention‐to‐treat approach, 68.9% of studies were at low risk of bias, 15.6% raised some concerns and 15.6% were at high risk of bias (seven studies). The domains most frequently associated with a high risk of bias were missing outcome data and selection of the reported results (Table S5). On the other hand, for analyses conducted according to the per‐protocol approach, 55.6% of studies were judged to be at low risk of bias, while 44.4% raised some concerns. In these cases, the most frequently implicated domains were missing outcome data and selection of the reported results (Table S5). Studies judged at high risk of bias in the ‘missing outcome data’ domain typically had, for example, high dropout rates, with limited information on the nature of the dropouts. Studies judged at high risk of bias in the ‘selection of the reported results’ domain showed uncertainty regarding the designation of primary outcomes and possible selective reporting of results.

### 3.2 Results of PRECIS‐2

#### 3.2.1 Overall Results

The average PRECIS‐2 score for all selected RCTs across all 10 domains was 2.93 (SD 1.30), indicating an overall balance between the explanatory and pragmatic levels of these studies. The study with the highest score had a score of 4.00 (an exercise‐based intervention conducted in Singapore, published in 2019) [36], and the study with the lowest score had a score of 2.13 (international study with nutritional intervention, published in 2020) [37]. The individual PRECIS‐2 scores for each study can be found in supplementary material (Table S4).

#### 3.2.2 PRECIS‐2 Domains

**FIGURE 2:**
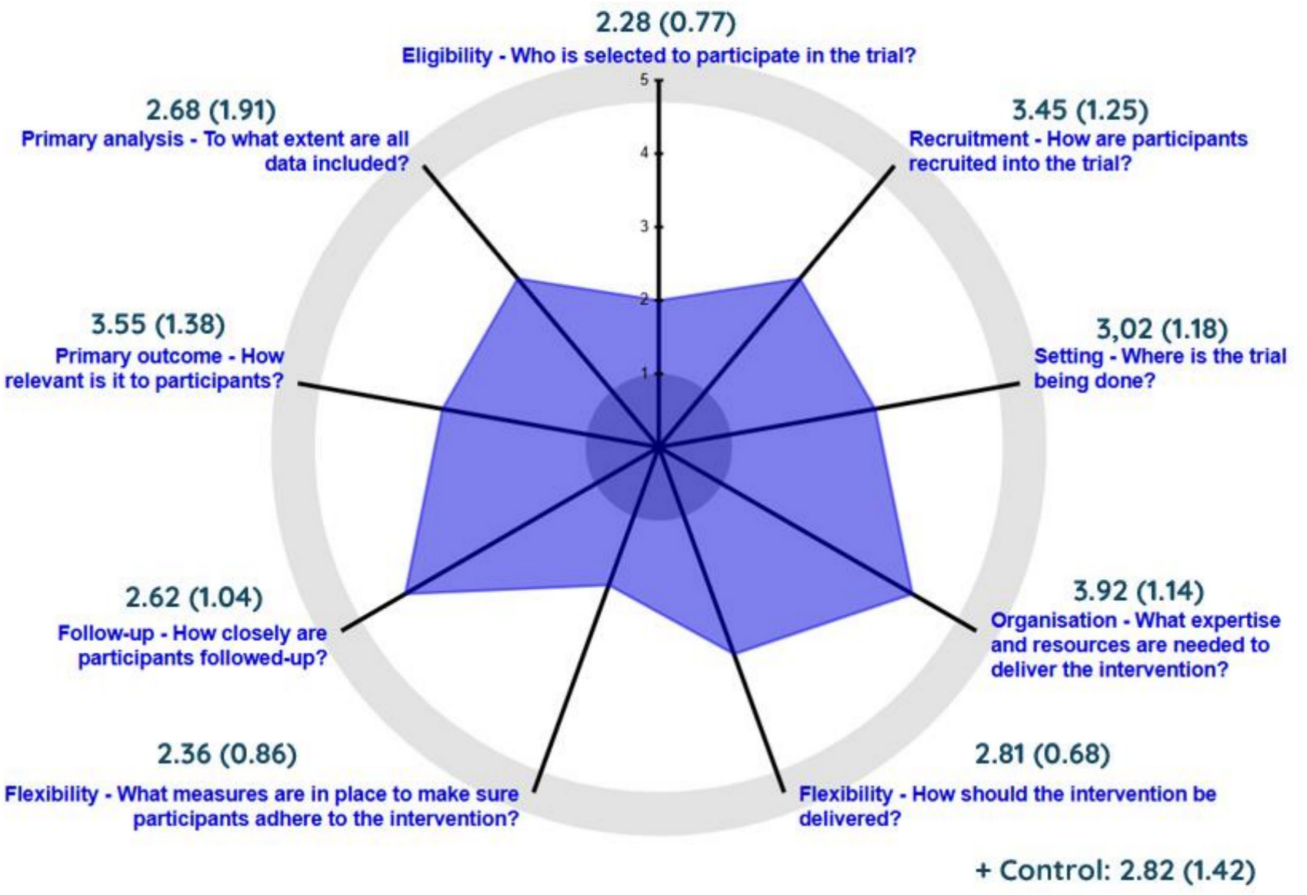
PRECIS‐2 results for the 54 RCTs included in the systematic review (mean ± standard deviation [SD], total score 2.93 ± 1.30).

Figure 2 shows the PRECIS‐2 wheel representing the average score for the 10 domains (9 PRECIS‐2 domains + control domain) of the 54 RCTs. The most pragmatic domains were organization (3.86 ± 1.10), recruitment (3.51 ± 1.20) and primary outcome (3.48 ± 1.33), followed by setting (2.94 ± 1.15), control (2.83 ± 1.45), flexibility—delivery (2.78 ± 0.66) and primary analysis (2.67 ± 1.90). The least pragmatic domains were follow‐up (2.52 ± 1.07), flexibility—adherence (2.45 ± 0.87) and eligibility (2.28 ± 0.79).

Inter‐rater reliability for PRECIS‐2 scoring was overall good to excellent for eight domains, with lower agreement observed for setting (ICC = 0.33) and flexibility‐delivery (ICC = −0.32) (Table S6).

#### 3.2.3 Subgroup Analysis

Subgroup analyses are presented in Tables 4 and 5. The geographical location of the studies, categorized by continent, was found to significantly influence the overall pragmatism score of the clinical trials (*p* = 0.049). Specifically, three PRECIS‐2 domains showed significant variation depending on the continent: ‘setting’ (*p* = 0.029), ‘follow‐up’ (*p* = 0.014) and ‘control’ (*p* = 0.042). In the ‘setting’ domain, studies conducted in Asia were more pragmatic, whereas those from America were more explanatory. For the ‘follow‐up’ domain, trials from Europe demonstrated higher levels of pragmatism, in contrast to those from America, which were more explanatory. The ‘control’ domain was rated as more pragmatic in studies from Asia and more explanatory in those grouped under the ‘Other’ category, which includes Oceania and global studies. Overall, studies conducted in Asia showed the highest levels of pragmatism.

**TABLE 4:** Subgroup analysis of PRECIS‐2 domain scores by geographical location and diagnostic criteria in the 54 included randomized controlled trials (RCTs).

	Geographical location of the study					Definition of sarcopenia – Diagnostic criteria			
PRECIS‐2 domains	Asia (Mean ± SD) *n* = 31 (57.4%)	Europe (Mean ± SD) *n* = 13 (24.1%)	America (Mean ± SD) *n* = 7 (13.0%)	Other (Mean ± SD) *n* = 3 (5.6%)	*p* One‐way ANOVA *Significant	EWGSOP definition (Mean ± SD) *n* = 24 (44.4%)	AWGS definition (Mean ± SD) *n* = 24 (44.4%)	Other (Mean ± SD) *n* = 6 (11.1%)	*p* One‐way ANOVA *Significant
Eligibility	2.23 (0.72)	2.31 (0.95)	2.57 (0.79)	2.00 (1.00)	0.693	**2.42 (0.83)**	2.33 (0.64)	**1.50 (0.84)**	0.031*
Recruitment	3.58 (1.17)	3.42 (1.24)	3.20 (1.48)	4.00 (1.41)	0.854	3.44 (1.29)	3.59 (1.18)	3.40 (1.14)	0.910
Setting	**3.23 (1.04)**	2.77 (1.17)	**1.50 (0.58)**	2.67 (1.53)	0.029*	2.57 (1.21)	3.33 (0.96)	2.60 (1.34)	0.064
Organization	4.00 (1.08)	3.73 (1.10)	4.00 (0.82)	3.00 (1.73)	0.499	3.67 (1.14)	4.00 (1.08)	4.00 (1.22)	0.631
Flexibility‐delivery	2.77 (0.62)	2.62 (0.77)	3.14 (0.69)	2.67 (0.58)	0.403	2.83 (0.76)	2.79 (0.59)	2.50 (0.55)	0.549
Flexibility‐adherence	2.54 (0.88)	2.40 (0.70)	2.00 (1.41)	3.00 (0.00)	0.588	2.44 (0.89)	2.45 (0.93)	2.50 (0.71)	0.995
Follow‐up	2.35 (0.75)	**3.27 (1.35)**	**1.50 (0.58)**	2.67 (1.53)	0.014*	2.74 (1.33)	2.35 (0.75)	2.40 (1.14)	0.519
Primary outcome	3.77 (1.26)	3.15 (1.34)	2.71 (1.60)	3.67 (0.58)	0.197	3.21 (1.44)	3.88 (1.19)	3.00 (1.10)	0.142
Primary analysis	2.65 (1.98)	3.00 (1.96)	2.00 (1.73)	3.00 (1.73)	0.728	2.50 (1.84)	2.79 (2.00)	2.83 (2.04)	0.851
Control	**3.30 (1.24)**	2.08 (1.44)	2.50 (1.76)	**2.00 (1.73)**	0.042*	**2.13 (1.49)**	3.30 (1.11)	**3.67 (1.51)**	0.005*
**Total**	**3.05 (1.28)**	2.86 (1.31)	**2.55 (1.36)**	2.82 (1.25)	0.049*	**2.77 (1.33)**	**3.11 (1.25)**	2.85 (1.35)	0.023*

*Note:* Subgroups with the highest pragmatism scores are highlighted in green, while those with the lowest pragmatism scores are highlighted in red.

Abbreviations: AWGS, Asian Working Group for Sarcopenia; EWGSOP, European Working Group on Sarcopenia Older People; PRECIS‐2, PRagmatic Explanatory Continuum Indicator Summary; SD, Standard deviation.

**TABLE 5:** Subgroup analysis of PRECIS‐2 domain scores by intervention types, study sample size, and study duration in the 54 included randomized controlled trials (RCTs).

	Intervention types						Study sample size			Study duration		
PRECIS‐2 domains	Exercises (Mean ± SD) *n* = 19 (35.2%)	Nutrition (Mean ± SD) *n* = 11 (20.4%)	Combined (Mean ± SD) *n* = 17 (31.5%)	Pharmacological (Mean ± SD) *n* = 4 (7.4%)	Other (Mean ± SD) *n* = 3 (5.6%)	*p* One‐way ANOVA *Significant	Smaller sample ≤ 75 people (Mean ± SD) *n* = 32 (59.3%)	Larger sample > 75 people (Mean ± SD) *n* = 22 (40.7%)	*p* Student's *t*‐test for independent samples *Significant	Shorter study ≤ 12 weeks (Mean ± SD) *n* = 28 (51.9%)	Longer study > 12 weeks (Mean ± SD) *n* = 26 (48.2%)	*p* Student's *t*‐test for independent samples *Significant
Eligibility	2.21 (0.79)	2.00 (0.63)	2.35 (0.70)	2.50 (1.29)	3.00 (1.00)	0.349	2.37 (0.66)	2.14 (0.94)	0.311	2.18 (0.67)	2.38 (0.90)	0.347
Recruitment	3.13 (1.68)	3.50 (0.76)	3.76 (0.83)	4.00 (1.41)	3.67 (1.15)	0.639	3.29 (1.30)	3.88 (0.93)	0.081	3.30 (1.06)	3.73 (1.32)	0.244
Setting	2.71 (1.21)	3.36 (0.92)	2.94 (1.30)	3.00 (1.41)	2.67 (0.58)	0.684	2.86 (1.25)	3.05 (1.02)	0.567	2.96 (1.18)	2.92 (1.14)	0.892
Organization	3.71 (1.21)	3.67 (1.22)	4.14 (0.95)	4.00 (1.41)	4.00 (NA)	0.828	4.04 (0.96)	3.53 (1.30)	0.202	3.95 (0.90)	3.76 (1.30)	0.577
Flexibility‐delivery	2.79 (0.63)	3.00 (0.63)	2.71 (0.77)	3.00 (0.00)	2.00 (0.00)	0.198	2.91 (0.69)	2.59 (0.59)	0.078	2.89 (0.74)	2.65 (0.56)	0.185
Flexibility‐adherence	2.40 (0.55)	2.44 (0.88)	2.75 (0.87)	1.33 (0.58)	NA	0.086	2.44 (0.98)	2.45 (0.69)	0.974	2.60 (0.91)	2.29 (0.83)	0.338
Follow‐up	2.64 (1.03)	2.36 (0.92)	2.93 (1.10)	1.75 (0.96)	1.67 (1.15)	0.154	2.64 (1.22)	2.37 (0.83)	0.386	2.30 (1.02)	2.76 (1.09)	0.159
Primary outcome	3.42 (1.61)	3.72 (1.19)	3.41 (1.18)	3.00 (1.41)	4.00 (1.00)	0.847	3.34 (1.45)	3.68 (1.13)	0.341	3.43 (1.40)	3.54 (1.27)	0.764
Primary analysis	2.63 (1.98)	2.73 (2.00)	2.59 (1.97)	3.25 (1.50)	2.33 (2.31)	0.975	2.56 (1.93)	2.82 (1.89)	0.631	2.79 (1.97)	2.54 (1.86)	0.637
Control	3.78 (0.81)	1.55 (1.04)	2.81 (1.38)	**1.00 (0.00)**	**4.33 (1.15)**	< 0.001*	3.00 (1.37)	2.57 (1.57)	0.315	3.00 (1.47)	2.64 (1.44)	0.376
**Total**	2.99 (1.35)	2.81 (1.27)	3.03 (1.25)	2.55 (1.33)	3.0 (1.35)	0.271	2.96 (1.32)	2.90 (1.28)	0.606	2.94 (1.29)	2.93 (1.32)	0.913

*Note:* Subgroups with the highest pragmatism scores are highlighted in green, while those with the lowest pragmatism scores are highlighted in red.

Abbreviations: PRECIS‐2, PRagmatic Explanatory Continuum Indicator Summary; SD, Standard deviation.

The definition of sarcopenia used also significantly influenced the overall pragmatism score (*p* = 0.023). Two domains were notably affected: ‘eligibility’ (*p* = 0.031) and ‘control’ (*p* = 0.005). The ‘eligibility’ domain was assessed as more pragmatic when the EWGSOP criteria were applied, while other definitions (e.g., FNIH) were associated with a more explanatory approach. Regarding the ‘control’ domain, studies using definitions other than AWGS and EWGSOP scored as more pragmatic, whereas those using EWGSOP were more explanatory. Overall, studies applying the Asian Working Group for Sarcopenia definition showed the highest levels of pragmatism.

In contrast, the type of intervention did not significantly impact the overall pragmatism score (*p* = 0.271). However, the ‘control’ domain again showed significant differences depending on the intervention type (*p* < 0.001). More specifically, the subgroup ‘other’ interventions (e.g., acupuncture) were associated with higher levels of pragmatism, while pharmacological trials were more explanatory. No other subgroup characteristics were associated with statistically significant differences, either in the overall pragmatism score or within any individual PRECIS‐2 domain.

#### 3.2.4 Sensitivity Analyses

##### 3.2.4.1 Original PRECIS‐2 Tool (Without ‘Control’ Domain)

When the control domain, which is not part of the PRECIS‐2 tool [27, 28, 29], was excluded from the analysis, the mean overall pragmatism score across the nine standard PRECIS‐2 domains was 2.95 (SD 1.28). The ranking of domains remained unchanged.

##### 3.2.4.2 Risk of Bias Assessment

Excluding the seven studies assessed as having a high risk of bias, the average PRECIS‐2 score across all 10 domains for the selected RCTs was 2.96 (SD 1.32) (Table S7).

## 4 Discussion

This systematic review is the first to comprehensively assess the level of pragmatism in interventional sarcopenia studies and identify gaps and potential rooms of improvement to enhance the clinical relevance and feasibility of future trials in the real world. The findings reveal an overall balance between explanatory and pragmatic features across the 54 RCTs included. These trials tended to be both pragmatic and explanatory, with an average PRECIS‐2 score of 2.93 out of 5 (where 1 is *highly explanatory* and 5 is *highly pragmatic*). This suggests that, while they remain limited in their generalizability to everyday clinical practice, some areas are already pragmatic [28]. The most pragmatic domains were organization, recruitment, and primary outcome. These domains reflected actual care conditions, both in terms of the resources mobilized and the relevance of the criteria assessed. Conversely, follow‐up, flexibility‐adherence and eligibility received the lowest scores and were considered less pragmatic and more explanatory, primarily due to excessive follow‐up, rigid adherence strategies and numerous exclusion criteria.

Of the included studies, those conducted in Asia and using the AWGS definition were significantly more pragmatic. These results may be explained by the nature of the interventions in this continent. Indeed, most of the proposed interventions (i.e., 65%) were exercise‐based, and while differences by type of intervention were not statistically significant, exercise programs generally allow for greater flexibility in areas such as control and eligibility. Notably, a significant difference was observed in the ‘control’ domain (*p* < 0.001), which varied according to intervention type (*p* < 0.001). Pharmacological trials tended to be more explanatory in this domain. They often relied on placebo controls or highly standardized conditions for regulatory and safety reasons. Because of the lack of approved pharmacological treatments for sarcopenia [15], a low proportion (7.4%) of pharmacological trials was identified. Indeed, most of the molecules currently evaluated are still in Phase I or II [15]. The small number of pharmacological trials makes it difficult to evaluate the level of pragmatism of this type of intervention. In contrast, non‐pharmacological interventions, such as acupuncture or other unclassified methods, more often used comparators aligned with usual care. Importantly, adding the ‘control’ domain to the PRECIS‐2 tool accounted for this difference, as recommended in the literature. This enabled a more detailed assessment of the extent to which control conditions reflect routine clinical practice.

RCTs remain at the top of the clinical research evidence pyramid thanks to their ability to establish a link between intervention and beneficial effects on symptoms or disease [38, 39, 40]. The strict nature of RCTs remains essential to enhance both the internal validity of the study and the safety of participants, but there is room for improvement in certain domains to bring clinical trials closer to patient reality and real‐life care conditions [23, 41]. The need for pragmatism may vary depending on the type of intervention. While pharmacological trials require controlled conditions to ensure safety and regulatory compliance, non‐pharmacological interventions can more easily incorporate pragmatic elements related to adherence or eligibility. This is especially true in sarcopenia research, where patients frequently present with multimorbidity [17, 42, 43] and polypharmacy [44, 45]. In sarcopenia research, individuals who have difficulty adhering to exercise programs, whether due to physical limitations or other reasons, or those with severe forms of the disease, such as clinically significant impairments in physical performance, major mobility limitations, or very low scores on physical performance assessments, are often excluded from clinical trials. In such contexts, explanatory trials that exclude complex or vulnerable populations may produce results that fail to translate into clinical effectiveness [23]. For example, an exercise‐based intervention may appear highly effective in an RCT setting with close supervision and strict adherence protocols, but its impact may diminish substantially in real‐world conditions where support is limited and long‐term engagement is uncertain.

To address this translational gap, sarcopenia intervention studies could adopt more pragmatic approaches. For example, and depending on the type of intervention, adherence can be better reflected by favouring intention‐to‐treat analyses, eligibility can be broadened by reducing exclusion criteria when ethically feasible, and follow‐up can be lightened by limiting the number of visits. These modifications could make the trials more representative of real‐life clinical practice and more pragmatic.

There are also alternatives that may present both strengths and limits. For example, quasi‐randomized controlled trials (quasi‐RCTs) could be seen as an alternative for conducting more pragmatic research, especially for non‐pharmacological interventions. Although they do not involve the strict randomization of conventional RCTs [46] and have a lower level of internal validity, quasi‐RCTs offer other advantages. They allow for the inclusion of broader patient populations, including those often excluded from traditional RCTs [46]. Furthermore, quasi‐RCTs prioritize assessing effectiveness over efficacy, which is usually evaluated under ideal conditions [46]. Their higher external validity and ability to generalize findings to the overall population make them valuable tools for evaluating interventions in complex healthcare environments that closely reflect real‐life patient settings [47]. These characteristics make quasi‐RCTs a worthwhile option when designing clinical trials for sarcopenia.

Furthermore, RCTs would gain from not being considered in isolation. It is important to combine their results with those of observational studies [48], which often have less restrictive inclusion criteria and better reflect the diversity and complexity of real‐world patients. Observational studies are important for evaluating safety outcomes and long‐term effects, which are not fully captured in RCTs [49]. Combining data from RCTs and observational studies provides a more comprehensive understanding of an intervention's efficacy in routine clinical settings [48, 49]. This integrated approach allows for more nuanced, contextualized decision‐making in clinical practice and public health policy [49].

The growing emphasis on real‐world inclusion is reflected in the guidelines issued by regulatory authorities. Organizations such as the European Medicines Agency (EMA) and the U.S. Food and Drug Administration (FDA) now emphasize the importance of using real‐world data and designing clinical trials that better reflect medical care in clinical practice. The FDA encourages broadening inclusion criteria and adopting more inclusive recruitment practices [50]. Similarly, the EMA recognizes the importance of real‐world evidence to generate complementary data under real‐life conditions [51]. These changes indicate an evolution toward patient‐centred research. Clinical trials are now designed to not only test efficacy but also address the real needs, conditions, and diversity of patients in real life.

## 5 Limitations and Strengths

This review has several limitations that warrant consideration. Although the evaluation was conducted with methodological rigour, the critical assessment of the pragmatism domains inherently involves a degree of subjectivity. To reduce this potential bias, two independent reviewers carried out the evaluation, and discrepancies were resolved through consensus. Importantly, inter‐rater reliability was good to excellent in 8 out of the 10 domains, which indicates that both reviewers generally evaluated the domains in a consistent manner. However, a few domains showed poor inter‐rater reliability, highlighting the inherent subjectivity of the PRECIS‐2 tool, even when standardized domain definitions are applied. Additionally, the PRECIS‐2 tool does not allow for the assignment of decimal scores, which restricts evaluations, especially for tests between two levels of pragmatism. Another limitation was the lack of data in several of the included studies, which prevented the scoring of some domains and may have led to an underestimation of the true level of pragmatism. In line with Zwarenstein et al.’s recommendations [29], such domains with missing or insufficient information were deliberately left blank rather than being estimated, to preserve the integrity of the assessment. Another limitation may be related to the limited number of databases used (MEDLINE, Embase and CENTRAL), which may have restricted the comprehensiveness of the review and reduced the likelihood of identifying trials published exclusively in other databases. Similarly, excluding grey literature may have introduced publication bias by omitting studies with negative or neutral results that were not published. While this approach aligns with Cochrane’s methodological recommendations, it remains a potential limitation to consider when interpreting our results. Despite these limitations, the study presents several important strengths. This is the first systematic review to specifically assess the level of pragmatism in sarcopenia intervention studies, thereby providing an original and valuable contribution to the field. Additionally, the analysis includes the ‘control’ domain, as recommended in the literature [29], thereby enhancing the comprehensiveness of the evaluation. One of the key strengths of this review is the systematic assessment of risk of bias using the Cochrane RoB 2.0 tool. Beyond simply describing the overall risk, we conducted a sensitivity analysis by excluding studies deemed to be at high risk of bias (*n* = 7) to determine whether these trials had an influence on the PRECIS‐2 results. Notably, excluding these studies did not change the average overall score, but it did change the ranking slightly (recruitment and primary outcome were reversed). The ranking of the other domains has not changed. Finally, this systematic review offers practical guidance for improving future clinical trial designs by identifying commonly low pragmatic domains and proposing concrete directions for their improvement.

## 6 Conclusion

This systematic review highlights the moderate level of pragmatism in sarcopenia intervention studies, positioning these trials along the pragmatic–explanatory continuum. By identifying key methodological shortcomings, particularly in relation to eligibility, delivery adherence and follow‐up, this work provides valuable insights to improve the design and real‐world relevance of future clinical trials, and in particular for non‐pharmacological interventions. More specifically, enhancing pragmatism could be achieved by broadening inclusion criteria, simplifying follow‐up, and favouring intention‐to‐treat analyses. These findings support the development of interventions that are more patient‐centred and easier to implement, contributing to better integration of evidence into clinical practice.

We consider this systematic review as a piece of evidence that can serve as a basis for developing practical recommendations. A valuable next step would be to establish, for example, a working group including researchers, trial designers, geriatricians, physiotherapists, dietitians and older adults living with sarcopenia. Such an initiative would ensure that future sarcopenia trials are informed not only by existing literature but also by expert consensus and patient perspectives, ultimately improving their relevance and impact.

## Author Contributions

C.B., D.S.R. and Z.B. contributed to the study development and design. C.B. and D.S.R. contributed to the bibliographic search, data analysis and interpretation, as well as the writing of the draft. C.B., Y.M.C. and D.S.R. contributed to study selection, while S.V.H. and Z.B. were responsible for data extraction. S.V.H. and Y.M.C. assessed the risk of bias. S.V.H., C.B. and D.S.R. contributed to the final analysis and manuscript preparation. All authors revised the article for important intellectual content and provided their final approval of the submitted manuscript. All authors read and agreed to the final version of the manuscript.

## Funding

SVH is supported by a fellowship from the FSR (Fond Spécial de la Recherche) at the University of Namur.

## Ethics Statement

This is a systematic review; for this study type, Ethics Committee approval is not required.

## Consent

For this type of study, formal consent is not required.

## Conflicts of Interest

The authors declare no conflicts of interest.

## Supporting information


**Table S1:** PRISMA 2020 Checklist.

**Table S2:** Search strategies.

**Table S3:** Excluded studies.

**Table S4:** Characteristics of the 54 included randomized controlled trials.

**Table S5:** Risk of bias assessment of included randomized controlled trials using the Cochrane RoB 2.0 tool.

**Table S6:** Results of Inter‐rater reliability for PRECIS‐2 scoring.

**Table S7:** Sensitive analysis for the 47 randomized controlled trials included in the systematic review.
