# Supplementary material for: How Pragmatic Are Sarcopenia Intervention Studies? A Systematic Review
Source: J Cachexia Sarcopenia Muscle. 2026 Jan 22;17(1):e70181. doi: 10.1002/jcsm.70181 (PMC12828071; doi:10.1002/jcsm.70181)
Supplement: Supplementary file 2 — Table S2: Search strategies. [file JCSM-17-e70181-s006.docx]

**How pragmatic are sarcopenia intervention studies? A systematic review.**

**Table S2.** Search strategies.

**Database: Ovid MEDLINE(R) ALL <1946 to March 08, 2024>**

Search Strategy:

1 Sarcopenia/ (10381)

2 sarcopeni*.ti,ab,kf. (18556)

3 or/1-2 (19343)

4 Randomized Controlled Trial/ (609689)

5 randomi*.ti,ab,kf. (852111)

6 RCT*.ti,ab,kf. (82162)

7 Random Allocation/ (107056)

8 Double-Blind Method/ (177714)

9 single-blind method/ (33298)

10 ((singl* or doubl* or trip* or trebl*) adj2 (blind* or mask* or conceal*)).ti,ab,kf. (204988)

11 exp clinical trial/ (990388)

12 Controlled Clinical Trial/ (95577)

13 ((clinic* or interventi*) adj2 (stud* or trial*)).ti,ab,kf. (867321)

14 or/4-13 (2107327)

15 3 and 14 (2172)

16 limit 15 to english language (2127)

17 Case Reports/ (2389282)

18 "Systematic Review"/ (254400)

19 exp "review"/ (3296879)

20 Meta-Analysis/ (196369)

21 protocol*.ti. (100028)

22 exp animals/ not humans.sh. (5201238)

23 or/17-22 (10798954)

24 16 not 23 (1336)

**Database: Embase**

Session Results

.......................................................

No. Query Results Results Date

#17. #14 NOT #15 1,286 11 Mar 2024

#16. #14 NOT #15 1,286 11 Mar 2024

#15. protocol:ti 99,675 11 Mar 2024

#14. #13 AND 'Article'/it 1,379 11 Mar 2024

#13. #1 AND #12 2,650 11 Mar 2024

#12. #2 OR #3 OR #4 OR #5 OR #6 OR #7 OR #8 OR #9 OR 1,850,819 11 Mar 2024

#10 OR #11

#11. 'controlled intervention':ab,kw,ti 2,537 11 Mar 2024

#10. 'controlled trial':ab,kw,ti 248,742 11 Mar 2024

#9. 'controlled study':ab,kw,ti 75,590 11 Mar 2024

#8. 'clinical intervention':ab,kw,ti 7,466 11 Mar 2024

#7. 'clinical study':ab,kw,ti 95,594 11 Mar 2024

#6. 'clinical trial':ab,kw,ti 318,847 11 Mar 2024

#5. rct*:ab,kw,ti 121,116 11 Mar 2024

#4. randomi*:ab,kw,ti 1,220,125 11 Mar 2024

#3. 'randomized controlled trial (topic)' 269,825 11 Mar 2024

#2. 'randomized controlled trial' 1,097,580 11 Mar 2024

#1. 'sarcopenia'/exp OR sarcopenia 32,320 11 Mar 2024

**Database: EBM Reviews - Cochrane Central Register of Controlled Trials <February 2024>**

Search Strategy:

1 Sarcopenia/ (895)

2 sarcopeni*.ti,ab,kf. (2145)

3 or/1-2 (2423)

4 randomi*.ti,ab,kf. (1039071)

5 RCT*.ti,ab,kf. (42860)

6 ((singl* or doubl* or trip* or trebl*) adj2 (blind* or mask* or conceal*)).ti,ab,kf. (334471)

7 ((clinic* or interventi*) adj2 (stud* or trial*)).ti,ab,kf. (345654)

8 or/4-7 (1193045)

9 3 and 8 (1481)

10 limit 9 to english language (1470)

11 protocol*.ti. (35934)

12 10 not 11 (1362)
