# Supplementary material for: How Pragmatic Are Sarcopenia Intervention Studies? A Systematic Review
Source: J Cachexia Sarcopenia Muscle. 2026 Jan 22;17(1):e70181. doi: 10.1002/jcsm.70181 (PMC12828071; doi:10.1002/jcsm.70181)
Supplement: Supplementary file 4 — Table S4: Characteristics of the 54 included randomized controlled trials. [file JCSM-17-e70181-s003.docx]

**Table S4.** Characteristics of the 54 included randomized controlled trials.

| References | Localization  Mono-multicentric | Sample | Study design | Diagnosis of sarcopenia | Intervention | PRECIS-2 score | Risk of bias (RoB 2.0) | Funding source |
| --- | --- | --- | --- | --- | --- | --- | --- | --- |
| Exercise-based intervention (19 RCTs) | | | | | | | | |
| Chen et al. Exp Gerontol 2018. [1] | Taiwan  Multicentric | *n sample*: 33  *Age sample* (years):  -Intervention group 66.7 ± 5.3  -Control group 68.3 ± 2.8  *Sex ratio*: only women | Randomized controlled trial  8 weeks + 4 weeks of detraining | AWGS, Chen et al., 2014 | **2 arms**:  -Kettlebell training group KT: n=17  -Control group CON: n=16  Intervention: kettlebell weight training with 60%–70% of 1 repetition maximum was conducted twice a week for 8 weeks. | 3.11 | Low | Not reported (NR) |
| Courel-Ibáñez et al. J Am Med Dir Assoc 2022. [2] | Spain  Multicentric | *n sample*: 22  *Age sample* (years): 87.1 ± 7.1 years  *Sex ratio*: 41.7%M - 58.3%F | Multicenter randomized controlled trial  24 weeks (long training period) and 4 weeks (short training period with 2 weeks of break) | Foundation for the National Institutes of Health diagnosis algorithm by Studenski et al. (2019) | **2 arms**:  -Training-Short Detraining (LT-SD) group; n= 11  -Short Training-Long Detraining (ST-LD) group: n=11  Intervention: 24 weeks of supervised Vivifrail training followed by 6 weeks of detraining | 3.33 | Low | This work was supported by the Autonomous Community of the Region of Murcia, Regional Program for the Promotion of Scientific and Technical Research (Action Plan 2018), Seneca Foundation-Agency of Science and Technology, Region of Murcia (ID: 20872/PI/18). Mikel Izquierdo was supported by a research grant PI17/ 01814 of the Ministerio de Economía, Industria y Competitividad (ISCIII, FEDER). |
| De Sá Souza et al. Int J Environ Res Public Health 2022. [3] | Brazil  Multicentric | *n sample*: 28  *Age sample* (years): 76.03 ± 6.69  *Sex ratio*: 35.7%M - 64.3%F | Randomized, placebo-controlled, parallel-group study | EWGSOP1, Cruz-Jentoft et al., 2010 | **2 arms**:  -Intervention group: n= 14  -Control group: n=14  Intervention: Resistance training 3x/ week | 2.17 | Low | This research was funded by Conselho Nacional de Desenvolvimento Científico e Tecnológico (CNPq: 165071/2015-0—Fellowship to ST, VD’A and Scholarship to HSS), Coordenação de Aperfeiçoamento de Pessoal de Nível Superior (CAPES), Fundação de Amparo à Pesquisa do Estado de São Paulo (FAPESP: 2016/00521-9—Research grant), and Associação Fundo de Incentivo à Pesquisa (AFIP). |
| Flor-Rufino et al. Arch Gerontol Geriatr 2023. [4] | Spain  Multicentric | *n sample*: 51  *Age sample*: 79.8 ± 7.4  *Sex ratio*: only women | Single (researcher)-blinded randomized controlled  6 months | EWGSOP1, Cruz-Jentoft et al., 2010 | **2 arms**:  -High-intensity resistance training HIRT: n = 24  -Control group: n = 27  Intervention: two weekly 65 min group sessions for six months. Each consisting of three parts: a 10-minute warm up, a 45-min HIRT circuit, and a 10-min cool down | 3.00 | Some concerns | This work was supported by University of Valencia [Grant number UV-19-INV_AE19] and Conselleria de Innovacion, ´ Universidades, Ciencia y Sociedad digital, Valencia, Spain [Grant number GV/2020/071]. |
| Hassan et al. Geriatr Nurs 2016. [5] | Australia  Multicentric | *n sample*: 42  *Age sample* (years): 85.9 ± 7.5  *Sex ratio*: 31%M - 69%F | Two-group controlled  trial design  6 months | EWGSOP1, Cruz-Jentoft et al., 2010 | **2 arms**:  -Exercise (EX) group: n=21  -Usual care control (CON) group: n=21  Intervention: twice weekly progressive resistance and balance training up to 50 h over a six-month period | 2.90 | Low | Funding support was given to the lead of the parent project, J. Hewitt, to compensate her for the time invested. The funds were from a small consultancy account held by T. Henwood. No conflicts of interest have arisen during or from this research, and authors have received no financial benefit from publication |
| He et al. Arch Gerontol Geriatr 2024. [6] | China  Multicentric | *n sample*: 113  *Age sample* (years):  -ST: 67.76 ± 5.47  -SDSG 66.87 ± 3.84  -CG 65.42 ± 3.97  Sex ratio: 51%M - 49%F | Double-blind and randomized trial  24 weeks | AWGS, Chen et al., 2016 | **3 arms**:  -Strength training ST: n=30  -Self-determined sequence exercise program group SDSG: n=34  -Control group CG: n=30  Intervention: The strength training regimen (ST): comprises five distinct exercises, aimed at enhancing both upper and lower body strength. The exercises are categorized into lower body exercises and upper body exercises. | 3.38 | Low | The Fundamental Research Funds for Central Universities: Number 135222026. |
| Huang et al. Front. Neurol 2023. [7] | China  Multicentric | *n sample*: 60  *Age sample* (years):  -TC group: 69.70 ± 5.05  -Control group: 72.14 ± 4.79  Sex ratio: 55%M - 45%F | Parallel randomized controlled trial.  12 weeks | AWGS, Chen et al., 2014 | **2 arms**:  -Tai Chi group: n=30  -Control group: n=30  Intervention: The Tai Chi group followed a 12-week programme, with 36 40-minute sessions, three times a week. Each session included a warm-up, 30 minutes of Tai Chi and a cool-down. Based on a simplified eight-style Tai Chi, the programme aimed to improve stability and weight transfer, with adjustments to protect the knees. The programme progressed in three phases, gradually increasing in difficulty. A coach supervised the participants, and breaks were recommended in case of fatigue. | 2.50 | Low | This work was supported by the Research Launch Project of the Fourth People’s Hospital Affiliated to Tongji University (Grant Number: sykyqd02001) and the Zhejiang Province excellent Young talents Fund Project of traditional Chinese Medicine (Grant Number: 2022ZQ004). |
| Li et al. Front Med 2022. [8] | China  Multicentric | *n sample*: 70  *Age sample* (years): 77.89 ± 10.38  *Sex ratio*: 43%M - 57%F | Parallel-group, analyst blinded, randomized controlled trial  24 weeks | AWGS, Chen et al., 2014 | **2 arms**:  -Ditangquan exercise group DG: n=35  -Control group CG: n=35  Intervention: 10 min of warm-up, 40 min of Ditangquan exercise, and 10 min of cooling down three times a week. | 2.89 | Low | This work was supported by the Three-Year Action Plan for the Development of TCM in Shanghai–Highland Construction for International Standardization of TCM [No. ZY (2021–2023)-0212]. |
| Liang et al. Sci rep 2020. [9] | China  Monocentric | *n sample*: 60  *Age sample* (years): 87.3 ± 5.4  *Sex ratio*: 57%M - 43%F | Single-center, two-arm, parallel-group and single-blind RCT  12 weeks | AWGS, Chen et al., 2014 | **2 arms**:  -Intervention group (12-week mixed exercise): n=30  -Control group (12-week resistance exercise): n=30  Intervention: received a mixed exercise program including balance and resistance exercise. | 3.67 | Low | This work is supported by the National Key R&D Program of China [2018YFC2002104]. Te sponsors played no role in the design, methods, data collection, analysis, or preparation of this work. |
| Moghadam et al. J Nutr Health Aging 2020. [10] | Iran  NR | *n sample*: 30  *Age sample* (years): 64.3 ± 3.5  *Sex ratio*: men only | Prospective parallel group, single-blind randomized, controlled trial with three arms  8 weeks | NR | **3 arms**:  -ET followed by RT E+R: n=10  -RT followed by ET R+E: n= 10  -Non-exercise control group C: n=10  Intervention:  RT program: leg extension, leg curl, bench press, lat pulldown, lateral raise, and abdominal crunch  ET program: cycling on a fixed-speed cycle ergometer (15min first week;30min last week) | 2.86 | Low | This study was supported by Faculty of Sports Sciences of Tehran University. |
| Piastra et al. Biomed Res Int 2018. [11] | Italy  Multicentric | *n sample*: 72  *Age sample* (years):  -Resistance: 69.9±2.7  -Postural: 70.0±2.8  *Sex ratio*: only women | Prospective parallel group, randomized, controlled trial with two arms  36 weeks | EWGSOP1, Cruz-Jentoft et al., 2010 | **2 arms**:  -Muscle reinforcement training group: n=35  -Postural training group: n=37  Intervention: Each session of the muscle reinforcement training was divided into three phases: (1) standing 15 warming up and  motor coordination exercises; (2) standing/on the ground  30 muscle toning at low/moderate intensity for diferent  muscular districts (primarily abdominal and both lower and  upper limbs) with low weight loads (0.5, 1, or 1.5Kg); (3)  cooling down 15 and relaxation/stretching of the muscle systems with specifc exercises. | 3.00 | Low | NR |
| Rezaei et al. BMC Sports Sci Med Rehabil 2024. [12] | Iran  Multicentric | *n sample*: 23  *Age sample* (years): 74.87 ± 4.58  *Sex ratio*: only men | Semi-experimental design (two groups in the form of a pretest and a post-test)  8 weeks | EWGSOP2, Cruz-Jentoft et al., 2019 | **2 arms**:  -TRX Suspension Training (TST) group: n=10  -Control group: n=9  Intervention: three sessions per week, about 60 minuntes per session including warm up and cool down. | 2.89 | High | This research did not have any source of funding. |
| Seo et al. Int J Environ Res Public Health 2021. [13] | South Korea  Multicentric | *n sample*: 27  *Age sample* (years): 71.5 ± 5.1  *Sex ratio*: only women | Randomized Controlled Trial  16 weeks | IWGS | **2 arms**:  -Resistance training intervention: n=14  -Control group: n=13  Intervention: body weight-based and elastic band RT, 3x/week, 60mins/session | 2.38 | Low | This work was supported by the Ministry of Education of the Republic of Korea and the National Research Foundation of Korea (NRF-2018S1A5A2A03035186). |
| Valdes-Badilla et al. BMC Public Health 2023. [14] | Chile  Monocentric | *n sample*: 44  *Age sample* (years): 73.26 ± 8.35  *Sex ratio*: only women | Randomized controlled trial, single-blind, repeated measures of parallel groups  12 weeks | EWGSOP2, Cruz-Jentoft et al., 2019 | **2 arms**:  -Elastic band group EBG: n=22;  -Group-based dance: GBD: n=22  Intervention:  Elastic bands group (40 minuts): upper limbs, lower limbs  The colors of the elastic bands (yellow, red, green, blue, black, silver, and gold), each corresponding to a certain range of endurance strength, were used to indicate training loads | 3.17 | Low | This research received no external funding |
| Wang et al. J Nutr Health Aging 2020. [15] | China  Monocentric | *n sample*: 121  *Age sample* (years): 87.8 ± 4.9  *Sex ratio*: 78%M - 22%F | Prospective clinical trial.  2 weeks | AWGS, Chen et al., 2014 | **2 arms**:  -Intervention group: n = 62  -Control group: n = 59  Intervention: mixed exercise program including aerobic, balance, and resistance exercises that were personally tailored. | 3.33 | Low | This work is supported by the National Key R&D Program of China [2018YFC2002104]. The sponsors played no role in the design, methods, data collection, analysis, or preparation of this work. |
| Wei et al. Int J Environ Res Public Health 2022. [16] | China  Multicentric | *n sample*: 98  *Age sample* (years):  -YR: 66.70 ± 4.10  -RT: 66.87 ± 3.84  -CG: 65.42 ± 3.97  Sex ratio: 49.0%M - 51.0%F | Double-blind, randomized controlled trial  24 weeks | AWGS, Chen et al., 2014 | **3 arms**:  -Mixed resistance training group with Yi Jin Jing exercises (YR): n=33)  -Resistance training group (RT): n=33  -Control group (CG): n=32  Intervention: The YR group consisted of 30 min of Yi Jin Jing exercise and 30 min of resistance training.  The RT group consisted of 60 min of resistance training. | 3.00 | Low | The Fundamental Research Funds for Central Universities (Number: 135222026). |
| Yuenyongchaiwat et al. Clin Gerontol 2023. [17] | Thailand  NR | *n sample*: 90  *Age sample* (years): 69.82 ± 5.8  *Sex ratio*: 31.1%M - 68.9%F | Single-blind randomized controlled trial  12 weeks | AWGS, Chen et al., 2020 | **3 arms**:  -No sarcopenia: n= 30  -Sarcopenia with control group: n=30  -Sarcopenia with intervention program: n = 30  Intervention: increase their physical activity by walking + resistive exercise + record their daily walking steps and exercise in the daily booklet | 2.67 | Low | This project is funded by National Research Council of Thailand (NRCT), contract no. NRCT5-RSA63010-04 |
| Yuenyongchaiwat et al. Eur J Phys Rehabil Med 2022. [18] | Thailand  Monocentric | *n sample*: 60  *Age sample* (years): 70.58 ± 6.10  *Sex ratio*: 27%M - 73%F | Empirical study, designed as a randomized controlled  trial  12 weeks | AWGS, Chen et al., 2020 | **2 arms**:  -Intervention group: n=30)  -Control group (n=30)  Intervention: encouraging walking ≥7500 steps daily for 5 days/week + resistance exercise, was assigned to be performed twice a week. | 3.57 | Low | This project is funded by National Research Council of Thailand (NRCT), contract no. NRCT5-RSA63010-04. |
| Zhu et al. Eur J Ageing 2019. [19] | China  Monocentric | *n sample*: 90  *Age sample* (years):  -TC: 88.8±3.7  -WBC: 89.5±4.4  -CON: 87.5±3.0  *Sex ratio*: only men | Prospective randomized controlled trial  8 weeks | AWGS, Chen et al., 2014 | **3 arms**:  -Tai Chi TC group: n=30  -Whole-body vibration WBV group: n=30  -Control (CON) group: n=30  Intervention:  Tai Chi: 5 days per week for 8 weeks. 40 min and included a 10-min warmup, 20-min practice, and 10-min relaxation. The exercise procedures included: (1) brachial rewinding; (2) left- and right-knee kyphosis step; (3) left and right mustangmane; (4) cloud hand; (5) left and right Golden Rooster; (6) right and left foot pedal; (7) right- and left-wing fnger tips; and (8) crossing hands.  Whole-body vibration: 5 days per week for 8 weeks.40 min and included a 10-min warmup, 20-min vibration exercise (5 groups/time, and 3 min/  group, with 1-min resting between groups), and 10-min  relaxation. | 2.80 | Low | This study was supported by research for early diagnosis indexes and intervention methods of sarcopenia (Grant No., 15BJZ39). |
| Nutrition-based intervention (11 RCTs) | | | | | | | | |
| Bauer et al. Aging Clin Exp Res 2020. [20] | Belgium, Germany, Ireland, Italy, Sweden, and the United  Kingdom.  Multicentric | *n sample*: 233  *Age sample* (years): 77.7 ± 6.9  *Sex ratio*: 34.6%M - 65.4%F | 13-week double-blinded, randomized, isocaloric placebo-controlled trial  13 weeks | EWGSOP1, Cruz-Jentoft et al., 2010 | **4 arms**:  -WP-MND_RCT1_2 servings of WP-MND: n=39  -WP-MND_RCT1_1 serving of WP-MND: n=57  -WP-MND_RCT2_2 servings of WP-MND: n=72  -WP-MND_RCT2_1 serving of WP-MND: n=46  Intervention: 2 parts of WP-MND (20 g whey protein and 3 g total leucine, 9 g carbohydrates, 3 g fat, 800 IU (20 µg) vitamin D3, 500 mg calcium, and a mixture of vitamins, minerals and fbers) - to be taken before the breakfast and before the lunch | 2.13 | Some concerns | This study was financially supported and study products were provided by Danone Nutricia Research, Nutricia Advanced Medical Nutrition. The funding source was involved at all stages of the study. |
| Bauer et al. J Am Med Dir Assoc 2015. [21] | Belgium, Germany, Ireland, Italy, Sweden, and the United Kingdom  Multicentric | *n sample*: 330  *Age sample* (years): 77 ± 7.81  *Sex ratio*: 38%M - 62%F | Multicenter, randomized, controlled, double blind, 2 parallel-group study  13 weeks | EWGSOP1 | **2 arms**:  -Active group (n=184): received a vitamin D and leucine-enriched whey protein nutritional supplement  -Control group (n=196): received an iso-caloric control product  Intervention: active product (per serving) = 20 g whey protein, 3 g total leucine, 9 g carbohydrates, 3 g fat, 800 IU vitamin D, and a mixture of vitamins, minerals, and fibers | 3.00 | High | This study was financially supported, and study products were provided by Nutricia Research, Nutricia Advanced Medical Nutrition. The funding source was involved at all stages of the study. The final interpretation of the study results, review, and decision to submit the manuscript was performed by independent researchers with no affiliation to the funding source |
| Bo et al. Clin Nutr 2019. [22] | China  NR | *n sample*: 60  *Age sample* (years):  -Intervention: 73.23 ± 6.52  -Control: 74.83 ± 5.94  *Sex ratio*: 45%M - 55%F | 6-month double-blind, randomized, placebo-controlled  Trial  6 months | EWGSOP1, Cruz-Jentoft et al., 2010 | **2 arms**:  -Intervention group: n=30  -Placebo group: n=30  Intervention: whey protein, vitamin D and vitamin E | 3.56 | Low | The present study was funded by Danone Institute Diet, Nutrition Research and Communication Grant (No. DIC2015-02). |
| Cramer et al. J Am Med Dir Assoc 2016. [23] | Europe and North America  Multicentric | *n sample*: 330  *Age sample* (years): 77 ± 71.81  *Sex ratio*: 38%M - 62%F | Prospective, randomized, double-blinded, controlled, 2-treatment parallel study design.  24 weeks | EWGSOP1, Cruz-Jentoft et al., 2010 | **2 arms**:  -Control ONS: n=165  -Experimental ONS: n=165  Intervention: 20 g protein,11 g fat, 36 g carbohydrate,1.5 g CaHMB, 499 IU vitamin D3 and other vitamins, minerals, and nutrients in varying amounts | 2.78 | Low | This clinical trial was funded by Abbott Nutrition (clinicaltrials.gov identifier:  NCT01191125). JTC is also supported by the US Department of Agriculture National  Institute of Food and Agriculture Hatch project, Accession Number: 1009500.  JTC had a significant financial interest in Abbott Nutrition as a paid consultant  whose responsibilities were to assist with clinical trial design, analysis, interpretation of data, and training key study personnel at all clinical trial sites. |
| Lin et al. Clin Nutr 2021. [24] | Taiwan  Monocentric | *n sample*: 56  *Age sample* (years): 73.1 ± 6.92  *Sex ratio*: 71.4%M - 28.6%F | Double parallel-group clinical prospective  cohort study  12 weeks | AWGS, Chen et al., 2014 | **2 arms**:  -Diet group: n=28  -Supp group: n=28  Intervention: provided with a sachet containing supplements in addition to their regular daily meals to achieve the recommended protein intake (each supplement sachet included 88 kcal, 12.8 g of protein (including 8.5 g of whey protein concentrate), 1.2 g leucine, 7.3 g carbohydrates, 0.8 g fat and 120 IU vitamin D per serving) | 2.90 | Low | The study was supported by a grant (900400110685) from SMAD Biotechnology Co., Ltd. (Taipei, Taiwan). |
| Nasimi et al. J Am Med Dir Assoc 2020. [25] | Iran  Multicentric | *n sample*: 66  *Age sample* (years): 79.05 ± 7.17  *Sex ratio*: 31.6%M - 68.4%F | 12-week randomized double-blind controlled trial  12 weeks | AWGS, Chen et al., 2014 | **2 arms**:  -Intervention group: n=33  -Control group: n=33  Intervention: 300 g, HMB-,vitamin D-, and vitamin C-fortified yogurt (3 g HMB, 1000 IU vitamin D, and 500 mg vitamin C) | 2.90 | Some concerns | NR |
| Rondanelli et al. Geriatrics 2018. [26] | Italy  Monocentric | *n sample*: 200  *Age sample* (years): 81.37 ± 7.06  *Sex ratio*: 35.9%M - 64.1%F | Randomized, placebo-controlled and double-blind, using a parallel  group design  4 weeks | EWGSOP1, Cruz-Jentoft et al., 2010 | **4 arms**:  -Placebo: n=50  -Melatonin: n=50  -Amino acids: n=50  -Amino acids+melatonin: n=50  Intervention:  Amino acid supplementation: 42.0% leucine, 14.0% lysine, 10.5% valine, 10.5% isoleucine, 10.5% threonine, 7.0% phenylalanine, and 5.5% other-once a day, every day for 4 weeks  Melatonin supplementation: Onemilligram -30 mions before going to sleep | 2.20 | High | This research received no external funding |
| Rondanelli et al. Nutrients 2022. [27] | Italy  Monocentric | *n sample*: 60  *Age sample* (years): 79.71 ± 4.84  *Sex ratio*: NA | Randomized (1:1), double-blind, placebo-controlled, parallel-group, 2-month clinical intervention study  8 weeks | EWGSOP1, Cruz-Jentoft et al., 2010 | **2 arms**:  -Intervention group: n=30  -Placebo group: n=30  Intervention: omega-3 fatty acid (500 mg, consisting of n 64.71% EPA, 29.41% DHA and the remaining 5.88%  omega-3 in general), leucine (2.5 g), probiotic LPPS23 (“30 Billion”, freeze dried) (OLEP) | 2.70 | Some concerns | This research was funded by Abiogen Pharma (funding number OLEP/01). |
| Takeuchi et al. Geriatr Gerontol Int 2019. [28] | Japan  Multicentric | *n sample*: 68  *Age sample* (years): 79.9 ± 6.2  *Sex ratio*: 39.7%M - 60.3%F | 8-week, multicenter, randomized, controlled, blinded outcome, two-cohort parallel group intervention trial  8 weeks | AWGS, Chen et al., 2014 | **2 arms**:  -Intervention group: n=35  -Control group: n=33  Intervention: BCAA and vitamin D supplementation: 10.0 g  protein (BCAA, 2500 mg) and 12.5 μg vitamin D, and was  200.0 kcal/125.0 mL | 3.30 | Some concerns | NR |
| Tamura et al. Nutrients 2023. [29] | Japan  Multicentric | *n sample*: 100  *Age sample* (years):  -PG: 82.1 ± 6.1  -ALA50/SFC: 80.1±6.7  -ALA100/SFC: 80.3±8.3  -ALA150/SFC 77.7±6.7  -ALA100/SFC 81.8±6.5  *Sex ratio*: 36%M - 64%F | Multicenter, double-blinded, randomized-controlled trial  12 weeks | EWGSOP1, Cruz-Jentoft et al., 2010 | **5 arms**:  -Placebo group: n=20)  -ALA 50/SFC 29 group: n=18  -ALA 100/SFC 29 group: n=22  -ALA 150/SFC 29 group: n=19  -ALA 100/SFC 57 group: n=21  Intervention:  ALA 50/SFC 29 group: 50 mg ALA phosphate and 29 mg sodium ferrous citrate (SFC) food intake group  ALA 100/SFC 29 group: 100 mg ALA phosphate and 29 mg SFC food intake group  ALA 150/SFC 29 group: 150 mg ALA phosphate and 29 mg SFC food intake group  ALA 100/SFC 57 group: 100 mg ALA phosphate and 57 mg SFC food intake group | 2.43 | Low | SBI Pharmaceuticals Co., Ltd. financially supported this study. The study foods (containing 5-aminolevulinic acid and sodium ferrous citrate) in this study were also provided by SBI Pharmaceuticals Co., Ltd. The journal’s Article Processing Charge, the fee for technical assistance in the launch and execution of the study, and the medical writing of the manuscript by EviPRO Co., Ltd. were also financially supported by SBI Pharmaceuticals Co., Ltd. |
| Yoshimura et al. Nutr J 2019. [30] | Japan  Monocentric | *n sample*: 49  *Age sample* (years): 79.8 ± 6.7  *Sex ratio*: 31.8%M - 68.2%F | Eight-week, two-parallel group, single-center, randomized, controlled, open-label, blinded  8 weeks | AWGS, Chen et al., 2014 | **2 arms**:  -Intervention group AminoL40: n=24  -Control group: n=25  Intervention: leucine-enriched amino acids containing jelly-type supplement: comprised 3 g of leucine 40% enriched essential amino acids and 9.7 g of carbohydrate | 2.90 | Low | NR |
| Combined (exercises+nutrition)-based intervention (17 RCTs) | | | | | | | | |
| AzevedoPinheiro et al. Fisioter Mov 2020. [31] | Brazil | *n sample*: 60  *Age sample* (years): 80.3 ± 6.8  *Sex ratio*: only women | Randomized clinical trial with a convenience sample  of older women  12 weeks | EWGSOP1, Cruz-Jentoft et al., 2010 | **3 arms**:  -Functional exercise group FEG: n=20  -Functional exercise and nutritional intervention group NIG: n=20  -Control group CG: n=20  Intervention:  FEG: was based on the Homebased Older People’s Exercise (HOPE) program, developed by Clegg et al. (2010) for frail older women.  NIG: was submitted to the same training characteristics and frequency as the FEG. However, given that the muscular hypertrophy process depends on nutritional status, a nutritionist delivered a lecture on the topic, encouraging protein intake of 20 to 35% of total calories, since the presence of essential amino  acids, primarily leucine, may enhance anabolic potential, influencing the protein synthesis needed to increase muscle mass | 2.80 | High | NR |
| Bernabei et al. BMJ 2022. [32] | Italy, Spain, France, Czech Republic, Poland, Germany, Netherlands, Finland, United Kingdom, Austria, and Iceland  Multicentric | *n sample*: 1519  *Age sample* (years): 78.9  ± 5.8  *Sex ratio*: 28%M - 72%F | Multicentre randomized  controlled trial  36 months | FNIH by Mclean et al. 2014 | **2 arms**:  -Multicomponent intervention: n=760  -Lifestyle education: n=759  Intervention: combination of moderate intensity physical activity with technological support and nutritional counselling. | 3.20 | High | This work was funded by a grant from the Innovative Medicines Initiative Joint Undertaking (IMI−JU 115621), which included in-kind support by member companies of the European Federation of Pharmaceutical Industries and Associations (SanofiAventis, Novartis, GlaxoSmithKline, Servier, Astellas Pharma, and Boehringer Ingelheim). The funder had no role in the study design, data collection, data analysis, data interpretation, writing of the report, or decision to submit the article for publication. |
| Chang et al. Clin Nutr 2021. [33] | Taiwan  Monocentric | *n sample*: 58  *Age sample* (years): 74.99  ± 5.85  *Sex ratio*: 22.9%M - 77.1%F | Randomized controlled trial using a parallel-group design  26 weeks | EWGSOP1, Cruz-Jentoft et al., 2010 | **2 arms**:  -Early intervention group: n = 29  -Delayed intervention group: n = 29  Intervention: Hospital based exercise program, 2x/ week follow by home exercise moderate-intensity exercise 5 or more days/ week. the early intervention group, exercise training and nutrition supplementation were administered first followed by the home-based program. | 3.22 | Some concerns | The study was made possible by the research funding of the Community and Geriatric Medicine Research Center, National Taiwan University Hospital, Bei-Hu Branch, Taipei, Taiwan. |
| Chiang et al. Foods 2021. [34] | Taiwan  Multicentric | *n sample*: 36  *Age sample* (years): 84.97  ± 6.17  *Sex ratio*: 82.9%M - 17.1%F | Randomized controlled trial  12 weeks | AWGS, Chen et al., 2014 | **3 arms**:  -Control group: n=12  -Milk group: n=12  -Soy milk group: n=12  Intervention:  Milk group: 2x200 mL milk (96 kcal, 5.2 g protein, 2.6 g fat, 12 g carbohydrates, 260 mg Ca, 620.9 mg leucine)  Soy milk group: 2x200ml (97.4 kcal, 6.4 g protein, 3.0 g fat, 11.2 g carbohydrates, 220 mg Ca, 458.6 mg leucine) | 3.30 | Some concerns | This research was funded by Taipei Veterans General Hospital Yuanshan Branch, Taipei, Taiwan (YSVH10609) and Formosa Produce Corporation, Taipei, Taiwan (109-6202-029-300). |
| da Cruz Alves et al. Nutrients 2022. [35] | Brazil  Multicentric | *n sample*: 34  *Age sample* (years): 71 ± 5.08  *Sex ratio*: only women | Randomized, double-blind, placebo-controlled  Trial  14 weeks | EWGSOP1, Cruz-Jentoft et al., 2010 | **2 arms**:  -Group Exercise and Fish Oil (EFO): n=17  -Group Exercise and Placebo (EP): n=17  Intervention: Capsules of fish oil (Eicosapentaenoic acid (EPA); 440 mg and Docosahexaenoic acid (DHA); 220 mg + resistance exercise program | 2.33 | Low | This research was funded by the São Paulo Research Foundation (FAPESP). Grant 14/50258-7. |
| Han et al. Orthopaedic surgery 2022. [36] | China  Monocentric | *n sample*: 200  *Age sample* (years):  -HMB+RT group: 78.3 ± 5.9  -HMB: 77.1 ± 6.5  -RT group: 78.5 ± 6.5  -Control: 76.8 ± 7.1  *Sex ratio*: 74.6%M - 25.4%F | Pragmatic, prospective observational study  3 months | AWGS, Chen et al., 2014 | **4 arms**:  -HMB+RT group: n=50  -HMB group: n=50  -RT group: n=50  -Control group: n=50  Intervention:  HMB group: supplemented HMB (3 g/day)  RT group: In the first stage, 60%–70% 1RM exercise intensity was used for eight to 12 repetitions in each set, with two to three sets of training each time and 3–5 min of rest between sets. In the second stage, 80%– 90% 1RM exercise intensity was used for 15 repetitions ineach set, two to three sets for each training, with 3–5 min of rest between sets | 3.00 | Some concerns | This work was supported by specific grants from the scientific and technological personnel training project of Tianjin Health Commission (No.ZC20209) and National  Natural Science Foundation of China (No.81902740) and Science and technology project of Tianjin Health Commission No.ZC20053). |
| Kim et al. JAGS 2012. [37] | Japan  Multicentric | *n sample*: 155  *Age sample* (years):  -Exercise + AAS: 79.5 ± 2.9  -Exercise: 79.0 ± 2.9  -AAS: 79.2 ± 2.8  -Health education: 78.7 ± 2.8  *Sex ratio*: only women | Randomized controlled trial  3 months | NR | **4 arms**:  -Exercises + amino acid group: n=38  -Exercises group: n=39  -Amino acid group: n=39  -Health education group: n=39  Intervention:  Exercises group: comprehensive physical fitness and muscle mass enhancement training program of moderate intensity was  provided for the participants in the exercise groups. The  exercise intervention consisted of 60-minute exercise  sessions held at the TMIG twice per week for 3 months.  Amino acid supplementation: Essential AAS was provided for the participants in the AAS groups every 2 weeks. Packets of powdered amino acid supplements =42.0% leucine, 14.0% lysine, 10.5% valine, 10.5% isoleucine, 10.5% threonine, 7.0% phenylalanine, and 5.5% other. | 2.44 | Low | The sponsors had no role in the design of this study, subject recruitment, baseline and post survey, development of the intervention program, data analysis, or preparation of the manuscript. |
| Li et al. Applied physiology, nutrition, and metabolism 2021. [38] | China  Multicentric | *n sample*: 241  *Age sample* (years):  -Nutr: 70.04 ± 3.98  -Ex: 73.73 ± 5.69  -Nutr+Ex: 71.52 ± 5.28  -Routine: 72.91 ± 6.29  Sex ratio: 41.4%M - 58.6%F | Randomized controlled trial  12 weeks | AWGS, Chen et al., 2014 | **4 arms**:  -Nutrition supplementation Nutr : n=61  -Physical exercise Ex : n=62  -Nutrition combined with exercise Nutr+Ex : n=59  -Routine consultation: n=59  Intervention:  Nutrition supplement: Whey protein powder (10 g) was taken 3 times daily with meals. EPA (300 mg), DHA (200 mg), and vitamin D3 (250 IU) were taken in the form of capsules, with 2 pills per time and 2 times daily at 30 min after breakfast and dinner.  Exercise: resistance training and outdoor activity | 3.44 | Some concerns | This work was funded by a Danone Institute China Diet Nutrition Research & Communication Grant (DIC2019-01) and the Central Health Care Research Project (W2017BJ23). The funding agencies were not involved in the study design, data collation and analysis, manuscript writing, or submission. |
| Meza-Valderrama et al. Arch Gerontol Geriatr 2024. [39] | Spain  Monocentric | *n sample*: 32  *Age sample* (years): 81.6 ± 9.3  *Sex ratio*: 25%M - 75%F | Randomized, double-blind, placebo-controlled trial with two parallel groups  12 weeks | EWGSOP2, Cruz-Jentoft et al., 2019 | **2 arms**:  -Intervention group: n=17  -Control group: n=15  Intervention: 3 g/day of Ca-HMB and participated in a 12- week resistance training program (3 sessions/week) | 3.60 | Low | The study received a three-year funding award from the Nutricia Research Foundation in November 2016 (Project No. 2017-14). The study protocol was registered in clinicaltrials.gov (NCT02679742) on February 9, 2016, and published in Maturitas in September 2016 (doi: 10.1016/j.maturitas.2016.08.019). The awarding organization had no role in the design, development, conduct, interpretation, manuscript writing, or any other phase of this study. |
| Monti et al. J Cachexia Sarcopenia Muscle 2023. [40] | Italy  Multicentric | *n sample*: 45  *Age sample* (years): 78.7 ± 5.9  *Sex ratio*: 24%M - 76%F | Randomized controlled trial  2 years | EWGSOP2, Cruz-Jentoft et al., 2019 | **2 arms**:  -Control group (Healthy Aging Lifestyle Education HALE): n = 21  -Intervention group (multiComponent intervention MCI): n = 24  Intervention: Performing two‐center and one home‐based session of mixed aerobic, strength and balance training per week and receiving nutritional counselling | 3.30 | Low | The present work was funded by a grant from the Innovative Medicines Initiative – Joint Undertaking (IMI-JU 115621) and by PRIN project ‘NeuAge’ (2017CBF8NJ_001, Narici). |
| Mori et al. J Nutr Health Aging 2022. [41] | Japan  Monocentric | *n sample*: 91  *Age sample* (years):  -RT: 77.6 ± 5.2  -PRO: 77.8 ± 4.5  -RT+PRO: 77.7 ± 3.3  *Sex ratio*: 15.7%M - 84.3%F | Randomized controlled trial  24 weeks (+24 weeks of de-training) | AWGS, Chen et al., 2014 | **3 arms**:  -Whey protein supplementation after resistance training (RT + PRO): n = 27  -Resistance training only (RT): n = 27  -Whey protein supplementation only (PRO): n = 27  Intervention:  PRO supplementation: 11.0 g of protein and 2,300 mg of leucine  RT program: exercises using a resistance elastic band and bodyweight resistance exercises | 3.10 | Low | The Dairy Products Health Science Council and the Japan Dairy Association, 2019. H.M. received a grant from the Dairy Products Health Science Council and the Japan Dairy Association. |
| Rondanelli et al. J Cachexia Sarcopenia Muscle 2020. [42] | Italy  Monocentric | *n sample*: 140  *Age sample* (years): 81 ± 6  *Sex ratio*: 27%M - 63%F ​ | Single-site, double-blind, randomized, controlled trial  8 weeks | EWGSOP1, Cruz-Jentoft et al., 2010 | **2 arms:**  -Experimental formula: n=70  -Isocaloric control formula: n=70  Intervention: A whey protein-based food for special medical purposes enriched with leucine and vitamin D (Fortifit®, Nutricia), twice per day; exercise sessions daily, 5x/ week | 2.67 | Low | This work was supported and sponsored by the Azienda di Servizi alla Persona of Pavia and the University of Pavia. The sponsor had no role in the design and conduct of the study, in the collection, management, analysis, and interpretation of the data or in the preparation, review, or approval of the manuscript. |
| Tokuda et al. J Am Nutr Assoc 2023. [43] | Japan  Multicentric | *n sample*: 54  *Age sample* (years):  -RE group: 79.0 (77.0, 80.0)  -RE+EAA group: 78.0 (77.0, 79.0)  -RE+EAA+TCC group: 79.0 (77.0, 80.3)  *Sex ratio*: 15%M - 85%F ​ | Open-label, pilot, randomized controlled trial  24 weeks (long training period) and 4 weeks (short training period) | AWGS, Chen et al., 2019 | **3 arms:**  -Resistance exercise: n=18  -Resistance exercise with essential amino acids supplementation (RE + EAA): n=18  -Resistance exercise with essential amino acids and tea catechins supplementation (RE+EAA+TCC): n=18  Intervention:  The exercise program: included a 20-min warming up exercise and 40-min RE. The RE program included exercises using a resistance elastic band and bodyweight resistance exercises. Elastic band REs included lower and upper skeletal muscle exercises.  EAA and TCC supplementation: The EAA supplement was provided to participants in the RE + EAA and RE + EAA + TCC groups. The EAA supplement powder containing 17.6 kcal of energy and 3,000 mg of EAAs (containing leucine 1,200 mg, valine 330 mg, isoleucine 320 mg) per serving was consumed with mineral water. The TCC supplement powder was provided to the participants in the RE + EAA + TCC group. The TCC supplement containing 19 kcal of energy and 540 mg of TCCs per serving was consumed with mineral water. | 3.30 | Low | The Foundation for Dietary Scientific Research, 2020. The funders had no role in study design, data collection, and analyses, in the writing of the manuscript, or in the decision to publish the results. |
| Wang et al. Nutrients 2022. [44] | China  Multicentric | *n sample*: 234  *Age sample* (years):  -Control group: 69.88 ± 3.29  -Nutrition group: 68.18 ± 3.93  -Exercise group: 69.72 ± 3.60  -Comprehensive group: 70.16 ± 4.32  Sex ratio: 17%M - 83%F | Randomized controlled trials  12 weeks | AWGS, Chen et al., 2019 | **4 arms:**  -Control group: n=54  -Nutrition group: n=58  -Exercise group: n=62  -Comprehensive group: n=60  Intervention:  The nutrition group: received dietary management information through an app. The app featured the ability to assess each participant’s diet and provide recommendations for adjustments, focusing on energy and protein intake, especially the high-quality protein, and give recommended recipes.  The exercise group: received exercise management information. The app evaluated the participant’s exercise status and recommended the amount of exercise, such as 40 to 60 min of moderate-to-high-intensity exercise (brisk walking and jogging) and resistance training (seated leg raises, static squat against a wall, dumbbell lifts, elastic bands, etc.) for 30 min, 3 days a week or more  The comprehensive group: received both dietary and exercise management information. | 2.88 | Some concerns | The current study was supported by the Young fund of Shanghai municipal health commission (20194Y0443); Dietary Nutrition Research and Education Foundation of Danone Nutrition Center (DIC2019-03); Outstanding young Talents in the three-year Plan of Shanghai municipal public health system (2020–2022) (GWV-10.3-YQ22); Academic leader in the three-year Plan of Shanghai municipal public health system (2020–2022) (GWV-10.2-XD18); Key disciplines in the three-year Plan of Shanghai municipal public health system(2020–2022) (GWV-10.1-XK11). |
| Yang et al. J Nutr Health Aging 2023. [45] | China  Multicentric | *n sample*: 34  *Age sample* (years):  -HMBG: 72.89 ± 7.02  -PG: 71.44 ± 5.22  *Sex ratio*: 35%M - 65%F | Randomized, double-blind, placebo-controlled trial  12 weeks | AWGS, Chen et al., 2019 | **2 arms:**  -HMB group HMBG: n=18  -Placebo group PG: n=16  Intervention: Nutritional supplementation, especially beta-hydroxy-betamethylbutyrate (HMB), a metabolite of leucine, which is an essential branched-chain amino acid with an anabolic role in the muscles + Resistance exercise training | 3.20 | Low | This work was supported by the Danone Dietary Nutrition Research and Education Fund [grant number DIC2019-10]. The supporting source had no involvement or restrictions regarding publication. |
| Zdzieblik et al. Br J Nutr 2015. [46] | Germany  NR | *n sample*: 60  *Age sample* (years): 72.2 ± 4.68  *Sex ratio*: only men | Randomized double-blind placebo-controlled study  12 weeks | EWGSOP1, Cruz-Jentoft et al., 2010 | **2 arms:**  -Treatment group TG (collagen peptide supplementation): n=30  -Placebo group PG: n=30  Intervention: Resistance training + 15 g of collagen peptides/d | 2.30 | Low | Part of the costs were paid by Gelita AG, Uferstraße 7, Eberbach, Germany. |
| Zhu et al. Age Ageing 2019. [47] | China  Multicentric | *n sample*: 113  *Age sample* (years): 73.78 ± 6.86  *Sex ratio*: 23%M - 77%F | Prospective parallel group, single-blind randomized  controlled trial  12 weeks | AWGS, Chen et al., 2014 | **3 arms:**  -Exercise intervention n=40  -Combined exercise and nutrition supplement intervention: n=36  -Waitlist control intervention: n=37  Intervention:  Exercise: Group exercises included 5–10 min warm-up and cool-down routine, 20–30 min chair-based resistance exercises using TheraBands, and 20-min aerobic exercises.  Combined: two sachets of Ensure NutriVigor daily [Each sachet (54.1 g powder) contains 231 calories, 8.61 g protein, 1.21 g β-hydroxy β-methylbutyrate, 130 IU vitamin D and 0.29 g omega-3 fatty acid]. | 3.38 | Low | This study is supported by grants from the Institute of Ageing, and the Centre for Nutritional Studies of the Chinese University of Hong Kong |
| Pharmacological intervention (4 RCTs) | | | | | | | | |
| Achison et al. J Cachexia Sarcopenia Muscle 2022. [48] | United Kingdom  Multicentric | *n sample*: 145  *Age sample* (years):  -Perindoprol/Leucine: 78.1 ± 5.5  -Perindopril/Leucine Placebo: 79.5 ± 6.6  -Perindopril placebo/Leucine: 78.6 ± 6.5  -Placebo/Placebo: 79.0 ± 5.7  *Sex ratio*: 46%M - 54%F | Placebo-controlled, parallel group, double-blind, randomized two-by-two factorial trial  12 months | EWGSOP1, Cruz-Jentoft et al., 2010 | **4 arms:**  -Perindoprol/Leucine : n=39  -Perindopril/Leucine Placebo : n=34  -Perindopril placebo/Leucine: n=33  -Placebo/Placebo: n=39  Intervention: Perindopril erbumine over encapsulated with a gelatine capsule packed with microcrystalline cellulose | 2.63 | Low | The LACE trial (project reference 13/53/03) is funded by the Efficacy and Mechanism Evaluation (EME) Programme, an MRC and NIHR partnership. The views expressed in this publication are those of the authors and not necessarily those of the MRC, NIHR or the Department of Health and Social Care. |
| Papanicolaou et al. J Nutr Health Aging 2013. [49] | Colombia, Peru, South Africa, Brazil, Sweden, Finland, Denmark, France, Spain, Mexico, Israel, Chile, New Zealand, United Kingdom, Hong Kong  Multicentric | *n sample*: 170  *Age sample* (years): 75.9 ± 6.9  *Sex ratio*: only women | Randomized, double-blind, parallel-arm, placebo controlled, multicenter  6 months | Baumgartner et al. (1998) | **2 arms:**  -MK-0773: n=81  -Placebo: n=89  Intervention: The dose for MK-0773 for this study was 50mg orally b.i.d | 2.78 | High | The design of the study protocols was the responsibility of Merck Sharp & Dohme corp., a subsidiary of Merck& co. in collaboration with various health authorities. All analyses were conducted by biostatisticians at Merck. |
| Rooks et al. J Am Geriatr Soc 2017. [50] | USA  Multicentric | *n sample*: 40  *Age sample* (years): 72 ± 5.5  *Sex ratio*: 53%M - 47%F | Randomized, double-blind, placebo controlled, parallel-arm, proof-of-concept study  24 weeks | EWGSOP1, Cruz-Jentoft et al., 2010 | **2 arms:**  -Intervention group bimagrumab: n=19  -Placebo: n=21  Intervention: Single intravenous infusion of bimagrumab 30 mg/kg on Day 1 + second dose on Day 57) | 2.25 | Some concerns | The study was designed by the Novartis Institutes of Biomedical Research (NIBR) and executed in collaboration with external principal investigators and their study sites. An independent contractor analyzed the data, and the NIBR study team was responsible for data interpretation and preparation of this manuscript. |
| Rooks et al. JAMA Netw Open 2020. [51] | 13 countries: USA, Australia, New Zealand, Singapore, Japan,etc. | *n sample*: 220  *Age sample* (years): 79.1 ± 5.3  *Sex ratio*: 39.4%M - 60.6%F | Double-blind, placebo-controlled, parallel-arm, multicenter, phase II randomized clinical trial  28 weeks | EWGSOP1, Cruz-Jentoft et al., 2010 | **2 arms:**  -Bimagrumab: n=115  -Placebo: n=68  Intervention: Bimagrumab 700 mg every 4 weeks | 2.50 | Low | This study was sponsored by Novartis Institutes for BioMedical Research, Cambridge, Massachusetts/Basel, Switzerland and Novartis Pharmaceuticals Corporation, East Hanover, New Jersey. The sponsor was involved in all aspects of the study, including in the design and conduct of the study; collection, management, analysis, and interpretation of the data; preparation, review, and approval of the manuscript; and decision to submit the manuscript for publication |
| Other intervention (3 RCTs) | | | | | | | | |
| Lu et al. Jama Netw open 2019. [52] | Singapore  Multicentric | *n sample*: 246  *Age sample* (years): 70.0 ± 4.7  -Active interventions: 69.76 ± 4.31  -Standard care: 71.00 ± 6.65  *Sex ratio*: 35.9%M - 64.1%F | Post hoc secondary analysis of a parallel-group randomized clinical trial  24 weeks | AWGS, Chen et al., 2014 | **5 arms:**  -Nutrition intervention: n=49  -Physical intervention: n=48  -Cognitive intervention: n=50  -Combined intervention: n=49  -Standard of care control: n=50  Intervention: Resistance and balance training, nutritional enhancement with a commercial oral nutrition supplement formula, cognitive training, a combination of the preceding 3 interventions | 4.00 | Some concerns | This work was supported by the National Medical Research Council (grant NMRC/1108/2007) and the Agency for Science Technology and Research (JCO grant 1434m00115).  The funders had no role in the design and conduct of the study; collection, management, analysis, and interpretation of the data; preparation, review, or approval of the manuscript; and decision to submit the manuscript for publication. |
| Min-Kyun Oh et al. J Gerontol A Biol Sci Med Sci 2020. [53] | Korea  NR | *n sample*: 45  *Age sample* (years): 79.05 ± 7.17  -Experimental group: 76.94 ± 9.43  -Control group: 81.15 ± 4.90  *Sex ratio*: 31.6%M - 68.4%F | Randomized controlled study with  blinded participant (to outcome) and researcher  6 months | AWGS, Chen et al., 2014 | **2 arms:**  -Experimental group: n=23  -Control group: n=22  Intervention: Antigravity treadmill (AGT) and conventional rehabilitation. Experimental group received AGT for 20 minutes  as well as standardized rehabilitation treatment for 30 minutes. | 2.44 | Low | None declared |
| Soares Mendes Damasceno et al. J Aging Res 2019. [54] | Brazil  NR | *n sample*: 53  *Age sample* (years): 68.7 ± 7.13  -Group 1: 72 ± 7.9  -Group 2: 63.5 ± 3.3  -Group 3: 67.4 ± 7.7  *Sex ratio*: NA | Quantitative experimental blind longitudinal study  8 weeks | EWGSOP1, Cruz-Jentoft et al., 2010 | **3 arms:**  -G1 (n=11): composed of older people with sarcopenia who received acupuncture intervention  -G2 (n=4): composed of older people with sarcopenia who did not receive intervention  -G3 (n=12): composed of older people without sarcopenia who did not receive acupuncture intervention  Intervention: 24 acupuncture sessions 3x/ week | 2.63 | High | NR |

**References:**

1. Chen, H.T., et al., *Effects of 8-week kettlebell training on body composition, muscle strength, pulmonary function, and chronic low-grade inflammation in elderly women with sarcopenia.* Exp Gerontol, 2018. **112**: p. 112-118.

2. Courel-Ibanez, J., et al., *Impact of Tailored Multicomponent Exercise for Preventing Weakness and Falls on Nursing Home Residents' Functional Capacity.* J Am Med Dir Assoc, 2022. **23**(1): p. 98-104 e3.

3. de Sa Souza, H., et al., *Resistance Training Improves Sleep and Anti-Inflammatory Parameters in Sarcopenic Older Adults: A Randomized Controlled Trial.* Int J Environ Res Public Health, 2022. **19**(23).

4. Flor-Rufino, C., et al., *Resistance training of peripheral muscles benefits respiratory parameters in older women with sarcopenia: Randomized controlled trial.* Arch Gerontol Geriatr, 2023. **104**: p. 104799.

5. Hassan, B.H., et al., *Impact of resistance training on sarcopenia in nursing care facilities: A pilot study.* Geriatr Nurs, 2016. **37**(2): p. 116-21.

6. He, S., et al., *Self-determined sequence exercise program for elderly with sarcopenia: A Randomized controlled trial with clinical assistance from explainable artificial intelligence.* Arch Gerontol Geriatr, 2024. **119**: p. 105317.

7. Huang, D., et al., *Effects of 12 weeks of Tai Chi on neuromuscular responses and postural control in elderly patients with sarcopenia: a randomized controlled trial.* Front Neurol, 2023. **14**: p. 1167957.

8. Li, Z.R., et al., *Ditangquan exercises based on safe-landing strategies prevent falls and injury among older individuals with sarcopenia.* Front Med (Lausanne), 2022. **9**: p. 936314.

9. Liang, Y., et al., *A randomized controlled trial of resistance and balance exercise for sarcopenic patients aged 80-99 years.* Sci Rep, 2020. **10**(1): p. 18756.

10. Moghadam, B.H., et al., *The Effects of Concurrent Training Order on Satellite Cell-Related Markers, Body Composition, Muscular and Cardiorespiratory Fitness in Older Men with Sarcopenia.* J Nutr Health Aging, 2020. **24**(7): p. 796-804.

11. Piastra, G., et al., *Effects of Two Types of 9-Month Adapted Physical Activity Program on Muscle Mass, Muscle Strength, and Balance in Moderate Sarcopenic Older Women.* Biomed Res Int, 2018. **2018**: p. 5095673.

12. Rezaei, S., R. Eslami, and B. Tartibian, *The effects of TRX suspension training on sarcopenic biomarkers and functional abilities in elderlies with sarcopenia: a controlled clinical trial.* BMC Sports Sci Med Rehabil, 2024. **16**(1): p. 58.

13. Seo, M.W., et al., *Effects of 16 Weeks of Resistance Training on Muscle Quality and Muscle Growth Factors in Older Adult Women with Sarcopenia: A Randomized Controlled Trial.* Int J Environ Res Public Health, 2021. **18**(13).

14. Valdes-Badilla, P., et al., *Effectiveness of elastic band training and group-based dance on physical-functional performance in older women with sarcopenia: a pilot study.* BMC Public Health, 2023. **23**(1): p. 2113.

15. Wang, R., et al., *Effectiveness of a Short-Term Mixed Exercise Program for Treating Sarcopenia in Hospitalized Patients Aged 80 Years and Older: A Prospective Clinical Trial.* J Nutr Health Aging, 2020. **24**(10): p. 1087-1093.

16. Wei, M., et al., *Hybrid Exercise Program for Sarcopenia in Older Adults: The Effectiveness of Explainable Artificial Intelligence-Based Clinical Assistance in Assessing Skeletal Muscle Area.* Int J Environ Res Public Health, 2022. **19**(16).

17. Yuenyongchaiwat, K., C. Akekawatchai, and J. Khattiya, *Effects of a Pedometer-Based Walking Home Program Plus Resistance Training on Inflammatory Cytokines and Depression in Thai Older People with Sarcopenia: A Three-Arm Randomized Controlled Trial.* Clin Gerontol, 2023. **46**(5): p. 717-728.

18. Yuenyongchaiwat, K. and C. Akekawatchai, *Beneficial effects of walking-based home program for improving cardio-respiratory performance and physical activity in sarcopenic older people: a randomized controlled trial.* Eur J Phys Rehabil Med, 2022. **58**(6): p. 838-844.

19. Zhu, Y.Q., et al., *Tai Chi and whole-body vibrating therapy in sarcopenic men in advanced old age: a clinical randomized controlled trial.* Eur J Ageing, 2019. **16**(3): p. 273-282.

20. Bauer, J.M., et al., *Safety and tolerability of 6-month supplementation with a vitamin D, calcium and leucine-enriched whey protein medical nutrition drink in sarcopenic older adults.* Aging Clin Exp Res, 2020. **32**(8): p. 1501-1514.

21. Bauer, J.M., et al., *Effects of a vitamin D and leucine-enriched whey protein nutritional supplement on measures of sarcopenia in older adults, the PROVIDE study: a randomized, double-blind, placebo-controlled trial.* J Am Med Dir Assoc, 2015. **16**(9): p. 740-7.

22. Bo, Y., et al., *A high whey protein, vitamin D and E supplement preserves muscle mass, strength, and quality of life in sarcopenic older adults: A double-blind randomized controlled trial.* Clin Nutr, 2019. **38**(1): p. 159-164.

23. Cramer, J.T., et al., *Impacts of High-Protein Oral Nutritional Supplements Among Malnourished Men and Women with Sarcopenia: A Multicenter, Randomized, Double-Blinded, Controlled Trial.* J Am Med Dir Assoc, 2016. **17**(11): p. 1044-1055.

24. Lin, C.C., et al., *Effects of adequate dietary protein with whey protein, leucine, and vitamin D supplementation on sarcopenia in older adults: An open-label, parallel-group study.* Clin Nutr, 2021. **40**(3): p. 1323-1329.

25. Nasimi, N., et al., *A Novel Fortified Dairy Product and Sarcopenia Measures in Sarcopenic Older Adults: A Double-Blind Randomized Controlled Trial.* J Am Med Dir Assoc, 2021. **22**(4): p. 809-815.

26. Rondanelli, M., et al., *Is a Combination of Melatonin and Amino Acids Useful to Sarcopenic Elderly Patients? A Randomized Trial.* Geriatrics (Basel), 2018. **4**(1).

27. Rondanelli, M., et al., *Effectiveness of a Novel Food Composed of Leucine, Omega-3 Fatty Acids and Probiotic Lactobacillus paracasei PS23 for the Treatment of Sarcopenia in Elderly Subjects: A 2-Month Randomized Double-Blind Placebo-Controlled Trial.* Nutrients, 2022. **14**(21).

28. Takeuchi, I., et al., *Effects of branched-chain amino acids and vitamin D supplementation on physical function, muscle mass and strength, and nutritional status in sarcopenic older adults undergoing hospital-based rehabilitation: A multicenter randomized controlled trial.* Geriatr Gerontol Int, 2019. **19**(1): p. 12-17.

29. Tamura, Y., et al., *Efficacy and Safety of 5-Aminolevulinic Acid Combined with Iron on Skeletal Muscle Mass Index and Physical Performance of Patients with Sarcopenia: A Multicenter, Double-Blinded, Randomized-Controlled Trial (ALADDIN Study).* Nutrients, 2023. **15**(13).

30. Yoshimura, Y., et al., *Effects of a leucine-enriched amino acid supplement on muscle mass, muscle strength, and physical function in post-stroke patients with sarcopenia: A randomized controlled trial.* Nutrition, 2019. **58**: p. 1-6.

31. Pinheiro, H.A., et al., *Nutritional intervention and functional exercises improve depression, loneliness and quality of life in elderly women with sarcopenia: a randomized clinical trial.* Fisioterapia em Movimento, 2020. **33**.

32. Bernabei, R., et al., *Multicomponent intervention to prevent mobility disability in frail older adults: randomised controlled trial (SPRINTT project).* BMJ, 2022. **377**: p. e068788.

33. Chang, K.V., et al., *Effectiveness of early versus delayed exercise and nutritional intervention on segmental body composition of sarcopenic elders - A randomized controlled trial.* Clin Nutr, 2021. **40**(3): p. 1052-1059.

34. Chiang, F.Y., et al., *Effects of Milk or Soy Milk Combined with Mild Resistance Exercise on the Muscle Mass and Muscle Strength in Very Old Nursing Home Residents with Sarcopenia.* Foods, 2021. **10**(11).

35. da Cruz Alves, N.M., et al., *Randomised Controlled Trial of Fish Oil Supplementation on Responsiveness to Resistance Exercise Training in Sarcopenic Older Women.* Nutrients, 2022. **14**(14).

36. Han, Z., et al., *Effect of Resistance Training Combined with Beta-Hydroxy-Beta-Methylbutyric Acid Supplements in Elderly Patients with Sarcopenia after Hip Replacement.* Orthop Surg, 2022. **14**(4): p. 704-713.

37. Kim, H.K., et al., *Effects of exercise and amino acid supplementation on body composition and physical function in community-dwelling elderly Japanese sarcopenic women: a randomized controlled trial.* J Am Geriatr Soc, 2012. **60**(1): p. 16-23.

38. Li, Z., et al., *Effects of nutrition supplementation and physical exercise on muscle mass, muscle strength and fat mass among sarcopenic elderly: a randomized controlled trial.* Appl Physiol Nutr Metab, 2021. **46**(5): p. 494-500.

39. Meza-Valderrama, D., et al., *Supplementation with beta-hydroxy-beta-methylbutyrate after resistance training in post-acute care patients with sarcopenia: A randomized, double-blind placebo-controlled trial.* Arch Gerontol Geriatr, 2024. **119**: p. 105323.

40. Monti, E., et al., *Effects of a 2-year exercise training on neuromuscular system health in older individuals with low muscle function.* J Cachexia Sarcopenia Muscle, 2023. **14**(2): p. 794-804.

41. Mori, H. and Y. Tokuda, *De-Training Effects Following Leucine-Enriched Whey Protein Supplementation and Resistance Training in Older Adults with Sarcopenia: A Randomized Controlled Trial with 24 Weeks of Follow-Up.* J Nutr Health Aging, 2022. **26**(11): p. 994-1002.

42. Rondanelli, M., et al., *Improving rehabilitation in sarcopenia: a randomized-controlled trial utilizing a muscle-targeted food for special medical purposes.* J Cachexia Sarcopenia Muscle, 2020. **11**(6): p. 1535-1547.

43. Tokuda, Y. and H. Mori, *Essential Amino Acid and Tea Catechin Supplementation after Resistance Exercise Improves Skeletal Muscle Mass in Older Adults with Sarcopenia: An Open-Label, Pilot, Randomized Controlled Trial.* J Am Nutr Assoc, 2023. **42**(3): p. 255-262.

44. Wang, Z., et al., *Effects of Internet-Based Nutrition and Exercise Interventions on the Prevention and Treatment of Sarcopenia in the Elderly.* Nutrients, 2022. **14**(12).

45. Yang, C., et al., *Effects of Beta-Hydroxy-Beta-Methylbutyrate Supplementation on Older Adults with Sarcopenia: A Randomized, Double-Blind, Placebo-Controlled Study.* J Nutr Health Aging, 2023. **27**(5): p. 329-339.

46. Zdzieblik, D., et al., *Collagen peptide supplementation in combination with resistance training improves body composition and increases muscle strength in elderly sarcopenic men: a randomised controlled trial.* Br J Nutr, 2015. **114**(8): p. 1237-45.

47. Zhu, L.Y., et al., *Effects of exercise and nutrition supplementation in community-dwelling older Chinese people with sarcopenia: a randomized controlled trial.* Age Ageing, 2019. **48**(2): p. 220-228.

48. group, L.s., et al., *Effect of perindopril or leucine on physical performance in older people with sarcopenia: the LACE randomized controlled trial.* J Cachexia Sarcopenia Muscle, 2022. **13**(2): p. 858-871.

49. Papanicolaou, D.A., et al., *A phase IIA randomized, placebo-controlled clinical trial to study the efficacy and safety of the selective androgen receptor modulator (SARM), MK-0773 in female participants with sarcopenia.* J Nutr Health Aging, 2013. **17**(6): p. 533-43.

50. Rooks, D., et al., *Treatment of Sarcopenia with Bimagrumab: Results from a Phase II, Randomized, Controlled, Proof-of-Concept Study.* J Am Geriatr Soc, 2017. **65**(9): p. 1988-1995.

51. Rooks, D., et al., *Bimagrumab vs Optimized Standard of Care for Treatment of Sarcopenia in Community-Dwelling Older Adults: A Randomized Clinical Trial.* JAMA Netw Open, 2020. **3**(10): p. e2020836.

52. Lu, Y., et al., *Assessment of Sarcopenia Among Community-Dwelling At-Risk Frail Adults Aged 65 Years and Older Who Received Multidomain Lifestyle Interventions: A Secondary Analysis of a Randomized Clinical Trial.* JAMA Netw Open, 2019. **2**(10): p. e1913346.

53. Oh, M.K., et al., *Efficacy of Combined Antigravity Treadmill and Conventional Rehabilitation After Hip Fracture in Patients With Sarcopenia.* J Gerontol A Biol Sci Med Sci, 2020. **75**(10): p. e173-e181.

54. Soares Mendes Damasceno, G., et al., *Acupuncture Treatment in Elderly People with Sarcopenia: Effects on the Strength and Inflammatory Mediators.* J Aging Res, 2019. **2019**: p. 8483576.
