# Supplementary material for: How Pragmatic Are Sarcopenia Intervention Studies? A Systematic Review
Source: J Cachexia Sarcopenia Muscle. 2026 Jan 22;17(1):e70181. doi: 10.1002/jcsm.70181 (PMC12828071; doi:10.1002/jcsm.70181)

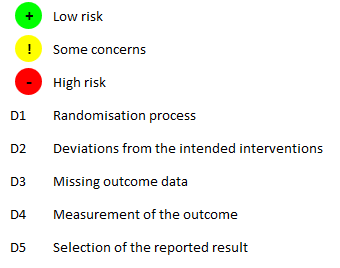

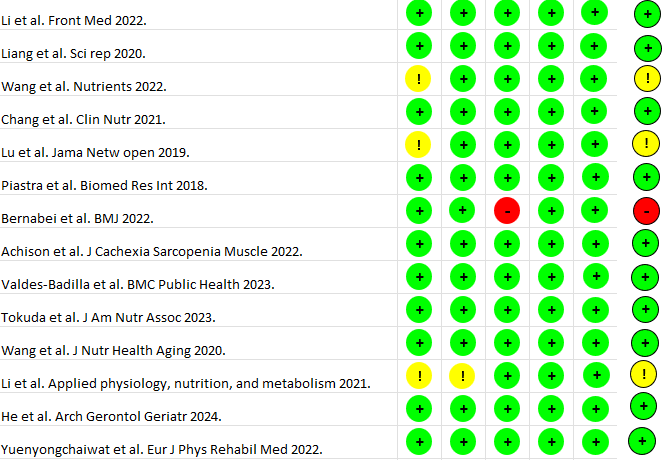
**
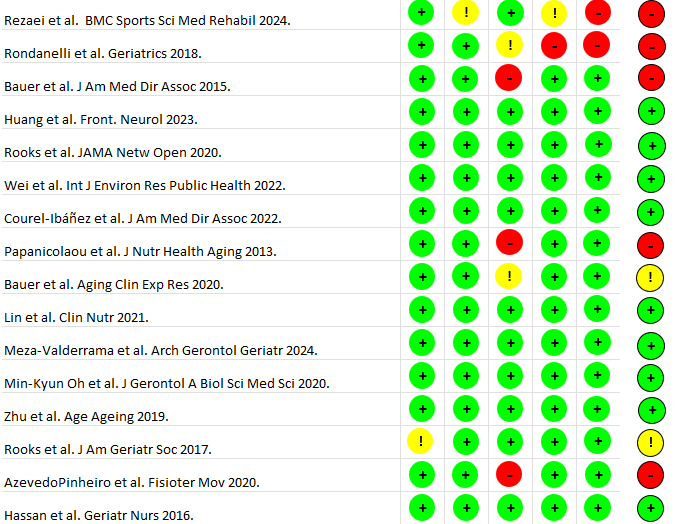

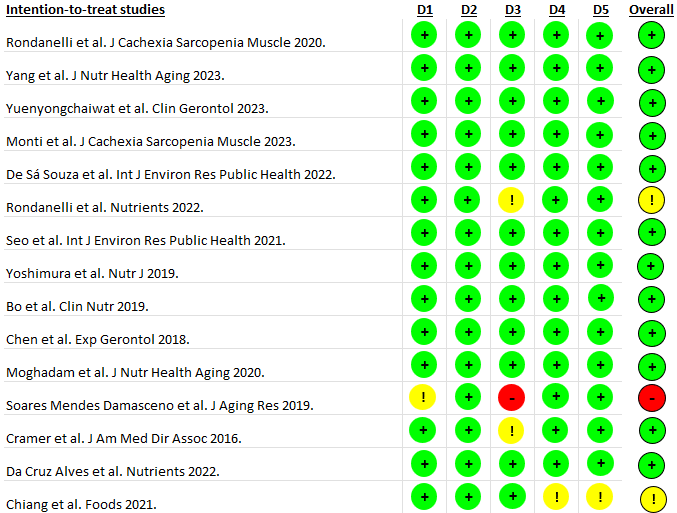
Table S5.** Risk of bias assessment of included randomized controlled trials using the Cochrane RoB 2.0 tool.


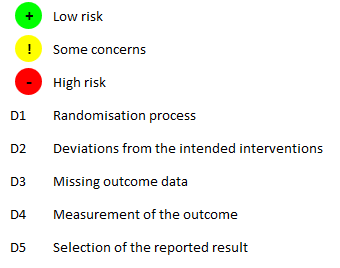

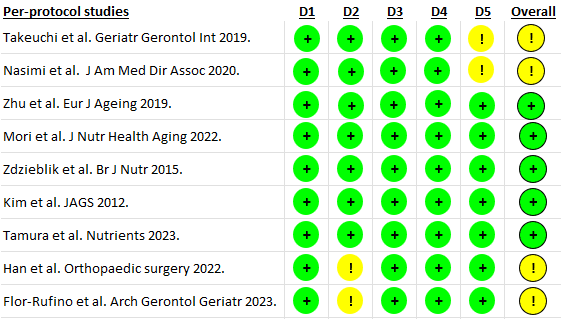

Supplement: Supplementary file 5 — Table S5: Risk of bias assessment of included randomized controlled trials using the Cochrane RoB 2.0 tool. [file JCSM-17-e70181-s005.docx]
