# Supplementary material for: How Pragmatic Are Sarcopenia Intervention Studies? A Systematic Review
Source: J Cachexia Sarcopenia Muscle. 2026 Jan 22;17(1):e70181. doi: 10.1002/jcsm.70181 (PMC12828071; doi:10.1002/jcsm.70181)
Supplement: Supplementary file 6 — Table S6: Results of Inter‐rater reliability for PRECIS‐2 scoring. [file JCSM-17-e70181-s001.docx]

**Table S6.** Results of Inter-rater reliability for PRECIS-2 scoring.

| PRECIS-2 domain | Intraclass correlation coefficients (ICC) | 95% CI | p-value | Interpretation (Koo and Li, 2016) |
| --- | --- | --- | --- | --- |
| *Eligibility* | 0.811 | 0.670 – 0.892 | <0.0001* | Good |
| *Recruitment* | 0.926 | 0.865 – 0.960 | <0.0001* | Excellent |
| *Setting* | 0.332 | -0.382 – 0.673 | 0.136 | Poor |
| *Organization* | 0.820 | 0.625 – 0.913 | <0.0001* | Good |
| *Delivery* | -0.321 | -1.513 – 0.284 | 0.821 | Poor |
| *Adherence* | 0.789 | 0.547 – 0.901 | <0.0001* | Good |
| *Follow-up* | 0.788 | 0.591 – 0.889 | <0.0001* | Good |
| *Primary outcome* | 0.912 | 0.846 – 0.949 | <0.0001* | Excellent |
| *Primary analysis* | 0.864 | 0.764 – 0.922 | <0.0001* | Good |
| *Control* | 0.970 | 0.947 – 0.983 | <0.0001* | Excellent |

*CI* Confidence interval ; *ICC* Intraclass correlation coefficients
