# Supplementary material for: How Pragmatic Are Sarcopenia Intervention Studies? A Systematic Review
Source: J Cachexia Sarcopenia Muscle. 2026 Jan 22;17(1):e70181. doi: 10.1002/jcsm.70181 (PMC12828071; doi:10.1002/jcsm.70181)
Supplement: Supplementary file 7 — Table S7: Sensitive analysis for the 47 randomized controlled trials included in the systematic review. [file JCSM-17-e70181-s004.docx]

**Table S7.** Sensitive analysis for the 47 randomized controlled trials included in the systematic review.


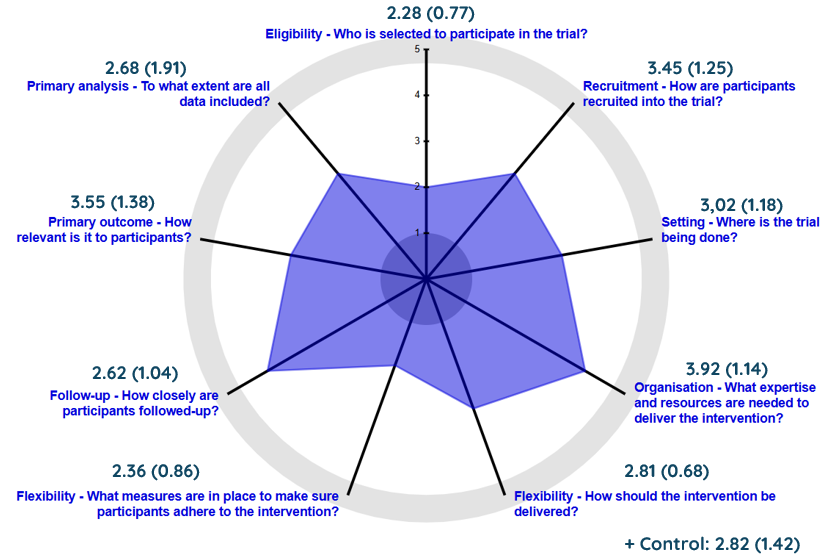


***Figure PRECIS-2 sensitive analysis for the 47 RCTs included in the systematic review (total score 2.96 ± 1.32)***

Out of the 54 randomized controlled trials (RCTs) identified by the systematic review, 7 trials were considered as having a high risk of bias and were excluded. PRagmatic Explanatory Continuum Indicator Summary (PRECIS)-2 tool was used to address the level of pragmatism in the 47 included RCTs [1-3]. PRECIS-2 includes nine domains: eligibility, recruitment, setting, organization, flexibility in delivery, flexibility in adherence, follow-up, primary outcome, and primary analysis. An additional domain, control, was incorporated specifically for this study. Although this is not formally part of the PRECIS-2 tool, Zwarenstein et al. [3], who were involved in the refinement of the original PRECIS into PRECIS-2 [4], have suggested the inclusion of a control-specific domain to better assess whether control conditions align with pragmatic principles, particularly in retrospective evaluations of clinical trials. This adaptation was made to enhance the tool’s ability to assess pragmatism in the context of sarcopenia trials.

The average PRECIS-2 score across all 10 domains for the 47 included RCT is 2.96 (SD 1.32). The figure below shows the PRECIS-2 wheel representing the average score for the 9 standard domains of the 47 RCTs (excluding the 7 studies identified as having a high risk of bias) and the control-specific domain. The most pragmatic domains are organization (3.92 ± 1.14), primary outcome (3.55 ± 1.38), and recruitment (3.45 ± 1.25), followed by setting (3.02 ± 1.18), control (2.82 ± 1.42), flexibility – delivery (2.81 ± 0.68), and primary analysis (2.68 ± 1.91). The least pragmatic domains are follow-up (2.62 ± 1.04), flexibility - adherence (2.36 ± 0.86), and eligibility (2.28 ± 0.77).

**References**

1. Loudon, K., et al., *The PRECIS-2 tool has good interrater reliability and modest discriminant validity*. J Clin Epidemiol, 2017. **88**: p. 113-121.

2. Loudon, K., et al., *The PRECIS-2 tool: designing trials that are fit for purpose*. BMJ, 2015. **350**: p. h2147.

3. Zwarenstein, M., et al., *PRECIS-2 for retrospective assessment of RCTs in systematic reviews*. J Clin Epidemiol, 2020. **126**: p. 202-206.

4. Loudon, K., *Making clinical trials more relevant: improving and validating the PRECIS tool for matching trial design decisions to trial purpose*. Trials, 2013. **14**.
